# Supplementary material for: The manufacture of AAV for gene therapy applications using a closed, semi-automated hollow-fiber bioreactor
Source: Mol Ther Methods Clin Dev. 2025 May 21;33(2):101496. doi: 10.1016/j.omtm.2025.101496 (PMC12167053; doi:10.1016/j.omtm.2025.101496)
Supplement: Document S1. Figures S1 and S2 and Tables S1–S9 [file mmc1.pdf]

OMTM, Volume *33*

Supplemental information

The manufacture of AAV for gene therapy applications using a closed, semi-automated hollow-fiber bioreactor

Adrien Soula, Florian Leseigneur, Amna Anwar, Bilal Ozdoganoglu, Jagan Gurung, Hamza Bhatti, Juline Guenat, Quentin Bazot, Majahar Sayed, Carolina Pinto Ricardo, Lily Li, Katerina Farukshina, Tony Bou Kheir, Hadi Mirmalek-Sani, Gregory Berger, Julie Kerby, Jonathan Appleby, and Michael Delahaye

SUPPLEMENTAL MATERIAL

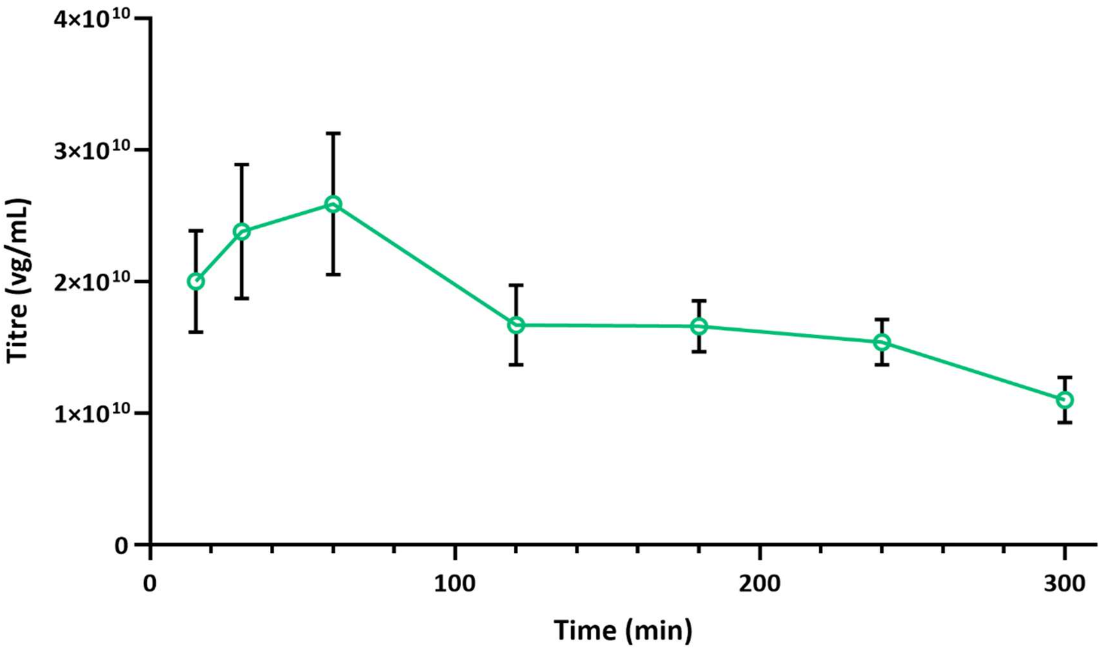

Figure S1: Quantum® lysis development. AAV2 viral genome titre resulting from time-course sampling, taken at 30-minute intervals across a 240-minute period to assess the impact of contact time on AAV2 liberation during the seventh Quantum® run. The error bars represent the standard deviation calculated from 9 measurements.

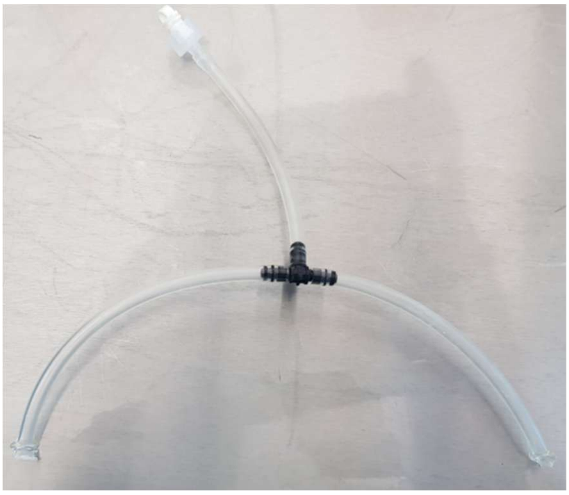

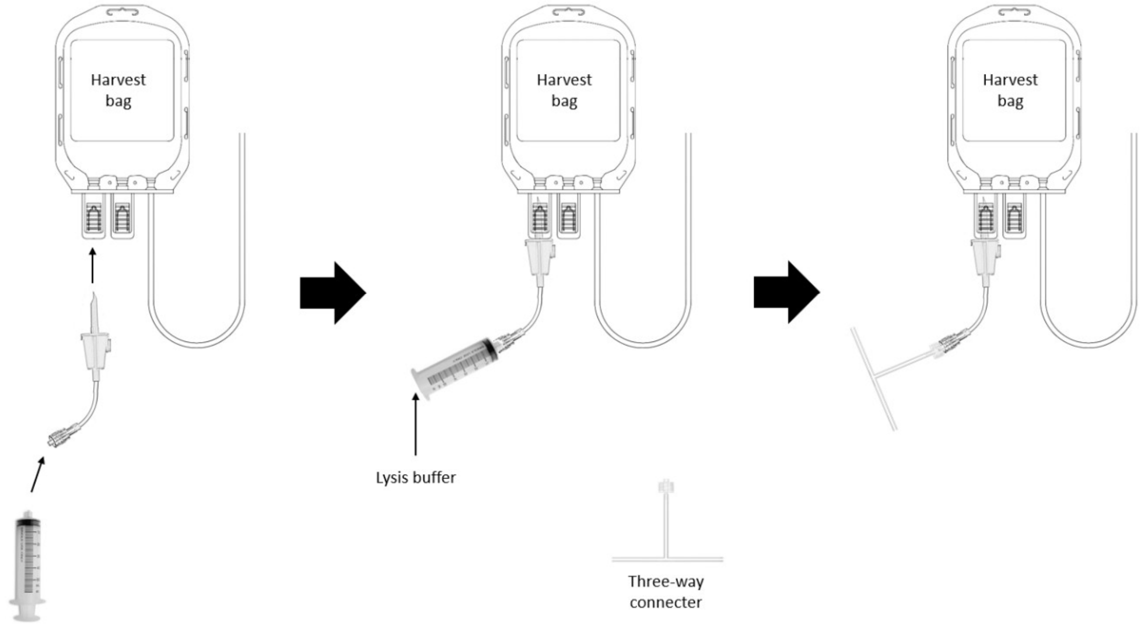

Figure S2: Fluid path adaptation. Use of three-way connecter to bypass the IC line, first a luer spike is used on the harvest bag, after lysis buffer addition, its luer end is used to seal the connection with the connecter.

Table S1: Quantum® Tasks settings for Day -1, set-up, priming and coating of the system. Reagent bag to contain fibronectin.

**Day**		***D-1***						
**Task**	**Task Settings**	**Load Cell Expansion Set**	**Prime**	**Remove IC Air**	**Remove EC Air**	**Coat Bioreactor**		
**Custom Task Grouping**		N/A	N/A	N/A	N/A	Step 1	Step 2	Step 3
		N/A	N/A	N/A	N/A	N/A	N/A	N/A
**IC inlet**		Default Settings	Default Settings	Inlet Source: Wash	Inlet Source: Wash	Reagent	Wash	None
**IC inlet rate**						10	10	0
**IC circulation rate**						100	100	20
**EC inlet**						None	None	Wash
**EC inlet rate**						0	0	0.1
**EC circulation rate**						30	30	30
**Outlet**						EC Outlet	EC Outlet	EC Outlet
**Rocker Control**						Stationary (0)	Stationary (0)	Stationary (0)
**Stop condition**						Empty Bag	IC Volume (22mL)	Manual
**Time**	**Extra info**	10 min	35 min	N/A	N/A	10 min	2 min	Overnight
**Necessary Volume (estimation)**		N/A	2L PBS	N/A	N/A	100 mL	22 mL	100 mL

Table S2: Tasks settings for Day 0, system conditioning and cell seeding. Cell bag to contain inoculum, media bags to contain media at 10% FBS.

**Day**		***D0***						
**Task**		**Inlet Line Wash-out**	**IC EC Washout**	**Condition Media**		**Load Cells with circulation**		
**Custom Task Grouping**		N/A	*Step 1*	*Step 2*	*Step 3*	Step 1	Step 2	Step 3
		N/A	CUSTOM TASK (1)			N/A	N/A	N/A
**IC inlet**	**Task Settings**	Source: Wash, Destination: Re-agent	IC Media	None	None	Cell	IC Media	IC Media
**IC inlet rate**			100	0	0	25	25	25
**IC circulation rate**			-17	100	100	139	139	-20
**EC inlet**			IC Media	IC Media	IC Media	None	None	None
**EC inlet rate**			148	0.1	0.1	0	0	0
**EC circulation rate**			-1.7	250	30	30	30	30
**Outlet**			IC & EC Outlet	EC Outlet	EC Outlet	EC Outlet	EC Outlet	EC Outlet
**Rocker Control**			In Motion	Stationary (0)	Stationary (0)	In Motion	In Motion	In Motion
			(-90, 180, 1)			(-90, 180, 1)	(-90, 180, 1)	(-90, 180, 1)
**Stop condition**			Exchange (2.5 IC, 2.5 EC)	Time (10 min)	Manual	Empty Bag	IC Volume (22 mL)	IC Volume (63 mL)
**Time**	**Extra info**	5 min	5 min	10 min	30-60 min	4 min	1 min	3 min
**Necessary Volume (estimation)**		N/A	800 mL	1 mL	<10 mL	100 mL	22 mL	63 mL

Table S3: Tasks settings for Day 0 to Day 5, cell attachment, cell feeding and media change prior to transfection. Media bags to contain media at 10% FBS, bag attached on IC media line for Step 8 of custom task 2 to contain media at 5% FBS.

**Day**		***D0 – D2***	***D2***	***D2 – D3***	***D3***	***D3 – D4***	***D4***	***D4 – D5***	***D5***
**Task**	**Task Settings**	**Attach Cells**	**Feed cells**						**Media change**
**Custom Task Grouping**		*Step 1*	*Step 2*	*Step 3*	*Step 4*	*Step 5*	*Step 6*	*Step 7*	*Step 8*
		CUSTOM TASK (2)							
**IC inlet**		None	IC media	IC media	IC media	EC media	EC media	EC media	IC media
**IC inlet rate**		0	0.2	0.4	0.8	0.8	1.2	1.6	2
**IC circulation rate**		0	-0.1	-0.1	-0.1	-0.1	-0.1	-0.1	0
**EC inlet**		IC media	IC media	IC media	IC media	EC media	EC media	EC media	None
**EC inlet rate**		0.2	0.2	0.4	0.4	0.4	0.4	0.4	0
**EC circulation rate**		30	50	100	100	100	100	100	100
**Outlet**		EC Outlet	EC Outlet	EC Outlet	EC Outlet	EC Outlet	EC Outlet	EC Outlet	Harvest
**Rocker Control**		Stationary (180)	Stationary (0)	Stationary (0)	Stationary (0)	Stationary (0)	Stationary (0)	Stationary (0)	Stationary (0)
**Stop condition**		Time (2,160 min)	Time (720 min)	Time (720 min)	Time (720 min)	Time (720 min)	Time (720 min)	Time (480 min)	Manual
**Time**	**Extra info**	36 h	12 h	12 h	12 h	12 h	12 h	8 h	4 h and up
**Necessary Volume (estima-tion)**		288 mL/day	576 mL/day	1,152 mL/day	1,728 mL/day	1,728 mL/day	2,304 mL/day	2,880 mL/day	500 mL

Table S4: Tasks settings for Day 5 to Day 7, transfection and cell feeding post-transfection with daily supernatant harvest. Reagent bag to contain transfection mix, media bags to contain media at 5% FBS, with 10% v/v of CDM4HEK293 from Step 5 of custom task 3.

**Day**		***D5***				***D5 – D6***	***D6***		***D6 – D7***
**Task**	**Task Settings**	**Add bag content**		**Incubate**		**Feed cells**	**Sup 24h**	**Feed cells**	
**Custom Task Grouping**		*Step 1*	Step 2	Step 3	Step 4	Step 5	Step 6	Step 7	Step 8
		CUSTOM TASK (3)							
**IC inlet**		Reagent	IC media	None	None	EC media	EC media	EC media	IC media
**IC inlet rate**		10	10	0	0	0.2	2	0.2	0.2
**IC circulation rate**		50	50	-2	-2	-0.1	0	-0.1	-0.1
**EC inlet**		None	None	IC media	IC media	EC media	None	EC media	IC media
**EC inlet rate**		0	0	2	2	1.8	0	1.8	2.3
**EC circulation rate**		300	300	300	300	300	300	300	300
**Outlet**		EC outlet	EC outlet	EC Outlet	EC Outlet	EC Outlet	Harvest	EC Outlet	EC Outlet
**Rocker Control**		In Motion	In Motion	In Motion	Stationary (0)	Stationary (0)	Stationary (0)	Stationary (0)	Stationary (0)
		(-90, 180, 1)	(-90, 180, 1)	(0, 180, 1800)					
**Stop condition**		Empty bag	IC volume (47mL)	Time (120 min)	Time (240 min)	Time (960 min)	Time (120 min)	Time (720min)	Time (624 min)
**Time**	**Extra info**	10 min	2 min	2 h	4 h	16 h	2 h	12 h	10.4 h
**Necessary Volume (estimation)**		180 mL	47 mL	240 mL	480 mL	2,880 mL/day	240 mL	2,880 mL/day	3,600 mL/day

Table S5: Tasks settings for Day 7 to Day 8, cell feeding post-transfection with daily supernatant harvest and wash prior cell detachment. Media bags to contain 5% FBS and 10% v/v CDM4HEK293, wash bag to contain PBS.

<table>
<tr><th>Day</th><th></th><th colspan="2">D7</th><th>D7 – D8</th><th colspan="4">D8</th></tr>
<tr><td>Task</td><td rowspan="13">Task Settings</td><td>Sup 48h</td><td colspan="2">Feed cells</td><td>Sup 72h</td><td colspan="3">Wash</td></tr>
<tr><td rowspan="2">Custom Task Grouping</td><td>Step 9</td><td>Step 10</td><td>Step 11</td><td>Step 12</td><td>Step 13</td><td>Step 14</td><td>Step 15</td></tr>
<tr><td colspan="7">CUSTOM TASK (3)</td></tr>
<tr><td>IC inlet</td><td>IC media</td><td>IC media</td><td>EC media</td><td>EC media</td><td>Wash</td><td>None</td><td>Wash</td></tr>
<tr><td>IC inlet rate</td><td>2.5</td><td>0.4</td><td>0.4</td><td>2.5</td><td>4</td><td>0</td><td>260</td></tr>
<tr><td>IC circulation rate</td><td>0</td><td>-0.1</td><td>-0.1</td><td>0</td><td>-2</td><td>0</td><td>-45</td></tr>
<tr><td>EC inlet</td><td>None</td><td>IC media</td><td>EC media</td><td>None</td><td>Wash</td><td>None</td><td>Wash</td></tr>
<tr><td>EC inlet rate</td><td>0</td><td>2.1</td><td>2.1</td><td>0</td><td>300</td><td>0</td><td>4</td></tr>
<tr><td>EC circulation rate</td><td>300</td><td>300</td><td>300</td><td>300</td><td>-3.5</td><td>0</td><td>2</td></tr>
<tr><td>Outlet</td><td>Harvest</td><td>EC Outlet</td><td>EC Outlet</td><td>Harvest</td><td>EC Outlet</td><td>EC Outlet</td><td>IC Outlet</td></tr>
<tr><td rowspan="2">Rocker Control</td><td>Stationary (0)</td><td>Stationary (0)</td><td>Stationary (0)</td><td>Stationary (0)</td><td>In Motion</td><td>Stationary (0)</td><td>In Motion</td></tr>
<tr><td></td><td></td><td></td><td></td><td>(-90, 180, 1)</td><td></td><td>(-90, 180, 1)</td></tr>
<tr><td>Stop condition</td><td>Time
(96 min)</td><td>Time
(720 min)</td><td>Time
(624 min)</td><td>Time
(96 min)</td><td>EC Volume
(500 mL)</td><td>Manual</td><td>IC Volume
(234 mL)</td></tr>
<tr><td>Time</td><td rowspan="2">Extra info</td><td>1.6 h</td><td>12 h</td><td>10.4 h</td><td>1.6 h</td><td>5 min</td><td>N/A</td><td>5 min</td></tr>
<tr><td>Necessary Volume (estimation)</td><td>240 mL</td><td>3,600 mL/day</td><td>3,600 mL/day</td><td>240 mL</td><td>500 mL</td><td>N/A</td><td>240 mL</td></tr>
</table>

Table S6: Tasks settings for Day 8, cell detachment and *in-situ* lysis. Reagent bag to contain TrypLE, media bag to contain media at 10% FBS for custom task 4. Custom task 5 to be performed according to Figure 2, lines will be sealed, and bypass will be performed during the waiting task (step 2 of custom task 5). Lysate will be recovered in the harvest bag.

**Day**		***D8***								
**Task**		**Harvest cells**				***In-situ* Lysis**			**Rapid IC washout**	**Unload Cell Expansion Set**
**Custom Task Group-ing**	**Task Settings**	Step 1	Step 2	Step 3	Step 4	Step1	Step 2	Step 3	N/A	N/A
		CUSTOM TASK (4)				CUSTOM TASK (5)			N/A	N/A
**IC inlet**		Reagent	Wash	None	IC media	Reagent	None	None	Reagent	Default Settings
**IC inlet rate**		30	30	0	400	40	0	o	260	
**IC circulation rate**		10	10	300	-70	-24	0	300	-45	
**EC inlet**		None	None	None	IC media	Reagent	None	Reagent	Reagent	
**EC inlet rate**		0	0	0	60	300	0	1.1	40	
**EC circulation rate**		30	30	30	30	-3.5	0	30	30	
**Outlet**		EC Outlet	EC Outlet	EC Outlet	Harvest	EC Outlet	EC Outlet	IC outlet	IC Outlet	
**Rocker Control**		In Motion	In Motion	In Motion	In Motion	In Motion	Stationary (0)	In Motion	In Motion	
		(-90, 180, 1)	(-90, 180, 1)	(-90, 180, 1)	(-90, 180, 1)	(-90, 180, 1)		(-90, 180, 1)	(-90, 180, 1)	
**Stop condition**		Empty Bag	IC Volume (22 mL)	Time (15 min)	IC Volume (378 mL)	EC Volume (500 mL)	Manual	60min	Exchange (1.3 IC, 0 EC)	
**Time**	**Extra info**	5 min	1 min	15 min	1 min	5 min	N/A	1 hour	5 min	5 min
**Necessary Volume (estimation)**		200 mL	22 mL	0mL	500 mL	800 mL	N/A	N/A	N/A	N/A

Table S7: Description of the first 4 Quantum® runs used to established cell expansion and vector production task settings.

Process step	1st run	2nd run	3rd run	4th run
**Seeding**	$200x10^6$ cells Obtained from 2D flasks	$200x10^6$ cells Obtained from 2D flasks	$40x10^6$ cells Obtained from thawed cryovials	$200x10^6$ cells Obtained from 2D flasks
**Expansion**	1 day attachment + 3 days expansion	1 day attachment + 3 days expansion	1.5 days attachment + 5.5 days expansion	1 day attachment + 3 days expansion
**Transfection**	N/A	2D historical parameters	N/A	2D historical parameters (PEI:DNA ratio changed from 3:1 to 1:1)
**Production**	N/A	2 days production	N/A	2 days production
**Harvest**	Release Adherent Cells and Harvest	Release Adherent Cells and Harvest	Release Adherent Cells and Harvest	Release Adherent Cells and Harvest
**Lysis**	N/A	Historical lysis (at $2x10^6$ cells/mL) performed in a 2D flask.	N/A	Historical lysis (at $2x10^6$ cells/mL) performed in a 2D flask.

Table S8: Description of the 3 Quantum® runs used to develop the in-situ lysis process step.

Process step	5th run	6th run	7th run
**Seeding**	$200x10^6$ cells<br>Obtained from 2D flasks	$40x10^6$ cells<br>Obtained from thawed cryovials	$40x10^6$ cells<br>Obtained from thawed cryovials
**Expansion**	1 day attachment + 4 days expansion	1.5 days attachment + 5.5 days expansion	1.5 days attachment + 5.5 days expansion
**Transfection**	Optimised parameters	Optimised parameters	Optimised parameters
**Production**	2 days production	2 days production	2 days production
**Harvest**	N/A	N/A	Release Adherent Cells, no Harvest
**Lysis**	Historical lysis, buffer introduced via reagent line	DoE at small scale on harvest material	POC lysis with bypass, time-course sampling

Table S9: Summary of yields obtained during the 3 Quantum® runs used to determine seeding density thus predicted density at transfection for the engineering runs.

Predicted density at transfection (cells/cm²)		Yield (total vg)						Lysis recovery (%)	Lysis & supernatants recovery (%)
		24h supernatant	48h supernatant	72h supernatant	Lysate	Flush post-lysis	Total		
**8th run**	**56K**	$1.49x10^{12}$	$2.39x10^{12}$	$8.71x10^{11}$	$5.62x10^{13}$	$2.94x10^{12}$	$6.39x10^{13}$	61.6	95.4
**10th run**	**83.3K**	$2.31x10^{11}$	$1.12x10^{11}$	$4.25x10^{10}$	$1.56x10^{14}$	$1.84x10^{12}$	$1.58x10^{14}$	98.7	99.0
**9th run**	**110K**	$2.22x10^{12}$	$5.00x10^{12}$	$2.07x10^{12}$	$3.18x10^{13}$	$6.57x10^{12}$	$4.76x10^{13}$	66.8	86.3
