# Supplementary material for: The manufacture of AAV for gene therapy applications using a closed, semi-automated hollow-fiber bioreactor
Source: Mol Ther Methods Clin Dev. 2025 May 21;33(2):101496. doi: 10.1016/j.omtm.2025.101496 (PMC12167053; doi:10.1016/j.omtm.2025.101496)
Supplement: Document S2. Article plus supplemental information [file mmc2.pdf]

Molecular Therapy
Methods & Clinical Development
Original Article

The manufacture of AAV for gene therapy applications using a closed, semi-automated hollow-fiber bioreactor

Adrien Soula,[1] Florian Leseigneur,[1] Amna Anwar,[1] Bilal Ozdoganoglu,[1] Jagan Gurung,[1] Hamza Bhatti,[1] Juline Guenat,[1] Quentin Bazot,[1] Majahar Sayed,[1] Carolina Pinto Ricardo,[1] Lily Li,[1] Katerina Farukshina,[1] Tony Bou Kheir,[1] Hadi Mirmalek-Sani,[1] Gregory Berger,[1] Julie Kerby,[1] Jonathan Appleby,[1] and Michael Delahaye[2]

[1]Cell and Gene Therapy Catapult, 12th Floor Tower Wing, Guy's Hospital, Great Maze Pond, London SE1 9RT, UK; [2]CCRM Nordic AB (SVB), Förändringens Gata 10, 431 53 Mölndal, Sweden

Adeno-associated viral (AAV) vectors have been established as a safe and effective delivery vehicle for gene therapy. However, current methods for AAV production using adherent approaches are suboptimal due to their reliance on a substantial number of plastic-based flasks, manual labor, and a significant manufacturing footprint. Consequently, a protocol for generating AAV2 was developed on the Quantum, a semi-automated closed hollow-fiber bioreactor platform. In this system, Human Embryonic Kidney 293T cells were successfully expanded and transfected to produce an average crude AAV2 titer of 4.92×10^{14} viral particles and 6.81×10^{13} viral genomes from 1.2 L of harvested cell lysate. The application of a standard AAV downstream process confirmed normal processability of the material. A cost of goods model comparing the Quantum bioreactor with the current standard HYPERStack36 and Corning CellSTACK 10-layer systems demonstrated that the Quantum bioreactor reduced the number of open steps by more than 40-fold, production time by up to 3.6-fold (HYPERStack36) and 7.5-fold (CellSTACK 10-layer), and costs by up to 2-fold (HYPERStack36) and 20.7-fold (CellSTACK 10-layer). Therefore, the Quantum bioreactor is an effective alternative to plastic flasks for the manufacturing of AAVs at both R&D and early translational scale, as it reduces production time, operating costs, and process risk.

INTRODUCTION

Gene therapy is a revolutionary medical technology that alters human genetic material to treat conditions caused by genetic abnormalities.[1,2] One of the most actively investigated approaches for the delivery of gene therapies is the use of recombinant adeno-associated virus (rAAV) as a viral vector.[3] AAV is a small (c.a. 25 nm), non-enveloped virus consisting of a protein shell surrounding and protecting a small, single-stranded deoxyribonucleic acid (DNA) genome of ~4.8 kilobases and can be engineered to deliver DNA sequences to target cells.[2–4] AAVs are excellent delivery vehicles for gene therapy due to their non-pathogenic nature; ability to attach to, and be internalized by, a wide variety of mammalian cells; transfer a genetic payload to the nucleus; and maintain expression from that genetic payload for a sustained duration.[4] Breakthroughs in AAV-based therapeutics include the first gene therapy, alipogene tiparvovec, licensed in Europe in 2012 to treat Lipoprotein Lipase Deficiency.[5] In 2017, the Food and Drug Administration (FDA) approved its first AAV-based gene therapy, voretigene neparvovec-rzyl, for Leber congenital amaurosis.[6] Since its launch, there have been five additional rAAV gene therapy products introduced to the market, with dozens more in development.[2] The rapid adoption of AAV-based therapeutics necessitates the development of robust manufacturing technologies to keep pace with both R&D and clinical demand.[2,3]

The dosing requirements of AAV therapeutics vary substantially. For example, the recommended dose for voretigene neparvovec-rzyl is approximately 1.5×10^{11} viral genomes (vg) per eye, whereas ongoing trials using AAV9 to treat Duchenne muscular dystrophy administer much higher dosages of up to 3.0×10^{14} vg per kilogram.[7,8] Traditionally, the production of rAAV involves transient transfection of plasmid DNA, encoding several elements of the vector genome, AAV Rep/Cap genes, Ad helper genes, and the Gene of Interest, into HEK293 cells.[3] The 293 cell line was developed for use as a tool to manufacture AAV by cloning into HEK cells certain additional helper genes required for vector formation.[9,10] In an attempt to boost productivity, the HEK293T derivative was generated, which incorporates the SV40 large T antigen (*SV40T*) gene, to stimulate Rep expression.[11] However, presence of the *SV40T* gene also creates a potential tumorigenic risk since AAV particles can theoretically package residual DNA encoding *SV40T*, which may inhibit p53 growth suppressive functions and thus pose a safety concern. Drug product made with the HEK293T cell line therefore requires additional safety testing, typically utilizing a PCR approach or vector

Received 20 November 2024; accepted 19 May 2025;
https://doi.org/10.1016/j.omtm.2025.101496.

Correspondence: Hadi Mirmalek-Sani, Cell and Gene Therapy Catapult, 12th Floor Tower Wing, Guy's Hospital, Great Maze Pond, London SE1 9RT, UK.
E-mail: hadi.mirmalek-sani@ct.catapult.org.uk

Published by Elsevier Inc. on behalf of The American Society of Gene and Cell Therapy.
This is an open access article under the CC BY license (http://creativecommons.org/licenses/by/4.0/).

sequencing approach prior to clinical release.[12] Adherent cell culture systems such as the CellSTACK 10-layer (CS10) and HYPERStack 36-layer vessels are widely used for medium-/small-scale AAV production, particularly during research and the translational development of new medicines.[13] HYPERStack, with its increased surface area, is designed for closed system scale-up from stacked plate products, delivering over double the yield relative to a stacked plate product of the same volumetric footprint.[13,14] Similarly, the CS10 culture vessel is a multi-layered flask; however, with 26 fewer layers it is less suitable for large-scale production.[15]

Both methods are manual, time-consuming, and have a large manufacturing physical footprint.[13–15] Despite efforts from associated manufacturers to provide closed fluid pathway solutions, issues may arise due to the inherent risk of contamination associated with the many open steps needed during flask-based manufacturing processes.[13] Further refinement of current production technologies should address these limitations, simplifying the generation of AAVs particularly at the scale typically required for studies conducted during the translation phase toward clinical trials. More recently, systems such as the iCELLIS from Cytiva, a fixed bed bioreactor, have shown promise in bridging the large-scale production needs when operated in combination with the use of adherent dependent cells.[16] Stirred tank bioreactors are increasingly recognized as the gold standard for manufacturing at a large scale, requiring adaptation of the packaging cell line to suspension production configuration.[13] A recent publication demonstrated a comparison of the three approaches from a cost perspective, comparing productivity and associated cost per dose across a production range of 200–1,000 L.[16] The findings demonstrated favorable output with respect to both stirred tank and fixed bed technologies, highlighting increased throughput and economies of scale. However, the demand for 200 L processes aligns with the higher end of requirement within clinical AAV demand, so a solution is required for the smaller scale needs, where dose requirements are lower, and production area is limited, such as in academic or hospital-associated facilities. A new method for the cost-effective production of biotherapeutic molecules is the Quantum hollow-fiber bioreactor. This is a semi-automated, closed, end-to-end dual-compartment bioreactor that produces a high yield of biological material.[17,18] The Quantum comprises a series of hydraulic pumps and valves that support a fluid circuit, with a gas transfer module for gas exchange.[17,18] Approximately 11,500 synthetic, semi-permeable, hollow fibers in the Quantum bioreactor provide up to 2.1 m^2 of surface area allowing unique and subtle control of the cell culture microenvironment.[17] These features provide versatility, efficiency, and scalability for expansion while reducing labor, material costs, and manufacturing footprint.[19] Previous studies have outlined how the Quantum system operates.[17] The Quantum bioreactor has been adopted to efficiently scale the production of various biological materials such as mesenchymal stem cells and T cells.[17–25] Additionally, the Quantum system has proven to be suitable for the production of lentiviral (LV) particles for gene therapy.[23] However, LV production poses a less complex challenge in that the target product, LV vector, is released from the packaging cell, making harvest and subsequent downstream processing less complex. AAV production, dependent on serotype, requires the liberation of AAV particles from within the packaging cell, thus adding complexity to the process.[26] Perhaps it is this challenge that has deterred investigators from exploring Quantum as an AAV production option to date. Compared to traditional flask-based methods to produce AAVs, the Quantum bioreactor offers the potential to significantly reduce operating costs, production time, and the number of open stages required during manufacture. In this article, we describe the development and performance of an AAV2 manufacturing process using the Quantum system and compare the performance relative to commonly used cell factory protocols.

RESULTS

Development of an AAV2 vector production process in Quantum

Baseline parameters (task settings) were received from Terumo, the system manufacturer, to perform basic cell expansion and AAV production using the Quantum bioreactor. The task settings consist of a sequential list of tasks, either already available from the system menu or manually entered by the user. We call the resulting sequence task settings, which can be shared in the form of a table listing settings from each process task. The final task settings (Tables S1–S6) used during engineering runs 1, 2, and 3 in this article are the optimized settings obtained after multiple iterations of development as described below.

Table S7 lists process step parameters of the first four development runs using the Quantum bioreactor to define an AAV2 vector production process. After the first proof-of-concept experiment where the cells were expanded but not transfected, a second run was performed transfecting the cells using historical transfection parameters, translated from 2D flasks. A third run was performed without transfection to focus on expansion of cryopreserved cells from cryovials. All task settings for those first three runs were provided by Terumo, whereas subsequent runs were adjusted as described in the final task settings in Tables S1–S6. A fourth run was performed with similar parameters as the second run, while reducing PEI to DNA ratio from 3:1 to 1:1. All cell harvests were performed using the task "Release Adherent Cells and Harvest" available from the Quantum software, whereas lysis was performed in a 2D flask outside of the bioreactor by diluting the cells to 2×10^6 cells/mL to align with historical 2D flask process. While the lysis from the second run yielded a total of 2.8×10^{13} vg, the fourth run yield was a total of 4.86×10^{13} vg, demonstrating improved production.

Following the initial investigation period, it was decided to optimize the transfection process, in parallel, in 2D flasks. The optimization consisted of four consecutive studies, which included two Design of Experiment (DoE) (Central Composite Design, face centered) designs, each followed by an associated experiment to confirm results from the associated DoE. The first DoE investigated the following parameters: PEI to DNA ratio, quantity of DNA per cell, and cell density at time of transfection. The second DoE investigated the plasmid ratio of the triple transfection. The confirmed optimized transfection

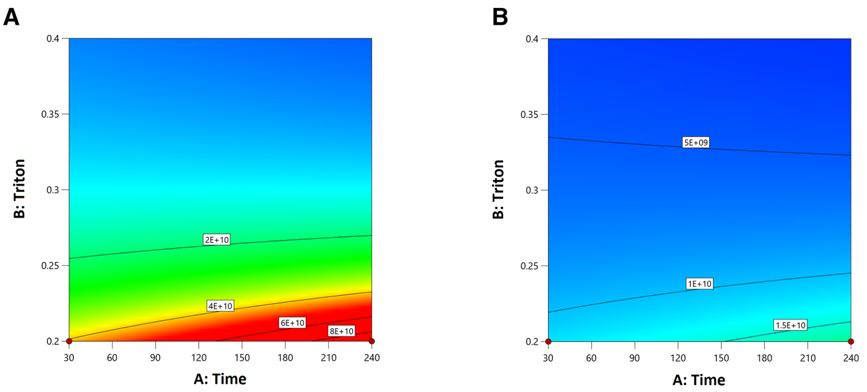

Figure 1. Lysis optimization study

Contour plots showing vg/mL output from a DoE study performed using cells harvested from the sixth Quantum experiment (model p value <0.05). Input variables were lysis buffer contact time (minutes), detergent concentration (%), and viable cells density.

(A) A vg/mL contour plot of the resulting model at 1×10^6 cells/mL.

(B) A vg/mL contour plot of the resulting model at 10×10^6 cells/mL. A dilution factor was applied to viral genomes titer in the modeling software.

parameters were then used in continued *in situ* lysis development for integration into Quantum production (data not shown).

Development of *in situ* lysis in Quantum

During manufacture AAV2 remains predominantly within the packaging cell. Since cell lysis was predicted to be the most challenging bioprocessing step associated with production in the Quantum, AAV2 was selected for development of the lysis step. A daily supernatant collection is also described; therefore, the protocol can be easily adapted for use with capsid types harvestable from the culture media. Table S8 lists process step parameters of the three development runs using the Quantum bioreactor to define the *in situ* lysis step. Recovery of the cell lysis material was anticipated to be a challenge because of the high cell density coupled with the restricted volume partitions in the Quantum system making recovery of lysate material challenging; therefore, a fifth experiment was performed, where lysis buffer was added to the intracapillary (IC) volume at the time of harvest. Historical lysis was applied, by the addition of Triton X-100 to reach a concentration of 0.5%, followed by 1 h contact time during which the lysis buffer was recirculated within the bioreactor. A total of 1×68.10^{12} total vg was recovered, considerably below the yields obtained during the first experiments with cells lysed outside of the system. Poor recovery confirmed the necessity of developing a novel process for *in situ* lysis.

Cells harvested from the sixth Quantum experiment were used to optimize lysis parameters (contact time from 30 min to 240 min, initial cell density from 1×10^6 to 10×10^6 cells/mL, and detergent [Triton X-100] concentration from 0.2% to 1.0%) via a DoE (Central Composite Design, face centered), and heatmaps from the resulting model are shown in Figure 1. A key finding from the DoE was the need to reduce the cell density of the harvested material. To address this, in a seventh run a bypass of the IC compartment was implemented before lysis to artificially extend its volume to 1.2 L (from the standard 175 mL), resulting in a more than 6-fold dilution of the harvest. This bypass utilized the harvest bag (included with the kit), which served as the final recipient of the lysate. The process is outlined in Figure 2, and the Quantum fluid path, before and after manual modification, is shown in Figure 3A.

The other finding from the DoE was an optimal lysis buffer concentration of 0.2% detergent (Triton X-100). The bypass method was tested during the seventh run in combination with time course sampling, taken at 30-min intervals across 240 min to assess the impact of contact time (Figure S1). A time point of 1 h was chosen, as no further improvement in recovery was observed beyond this time point. In a subsequent set of three runs (outlined in Table S9), cell density at transfection was optimized to improve productivity and recovery from the *in situ* lysis (task settings detailed in Tables S1–S6 were used). Predicted cell densities ranging 56–110K cells/cm^2 at transfection were examined. To calculate lysis recoveries, an extra flush of the system was performed after the *in situ* lysis using the "Rapid IC washout" task available from the software, changing parameters of this task to 2.5 times the IC volume used for flushing, into a cell inlet bag. Data from the 2D production process showed that the highest lysis recovery rate (98.7%, Figure 3B and Table S9) was achieved with a cell concentration of 83.3K cells/cm^2. This cell concentration was therefore used for further engineering runs in the Quantum.

Engineering runs—cell growth and metabolites

The focus of this study is the three Quantum runs that were performed using knowledge from all 10 runs of development and associated studies detailed previously (resulting parameters are detailed in the methods section and the task settings can be found in Tables S1–S6 in the supplemental material). Cell metabolites (glucose, lactate, glutamine, and ammonium) were monitored daily during the three engineering runs. Lactate measurements were used to predict cell growth within the Quantum bioreactor using a predefined predictive tool provided by Terumo, using lactate accumulation and perfusion rates as inputs. The predictive tool demonstrated close alignment for all three runs at approximately 5 billion cells (Figure 4A). Figure 4B illustrates metabolite trends over the 8-day process. Data show that cells perfused with fresh media prevented glucose depletion and lactate accumulation below or above critical levels. Steady utilization of glucose was observed until day 4, which subsequently accelerated toward day 8. Lactate and ammonium production inversely mirrored this trend, slowly increasing until day 4, after which production rate significantly increased. Glutamine fluctuated throughout the first 4-day period but

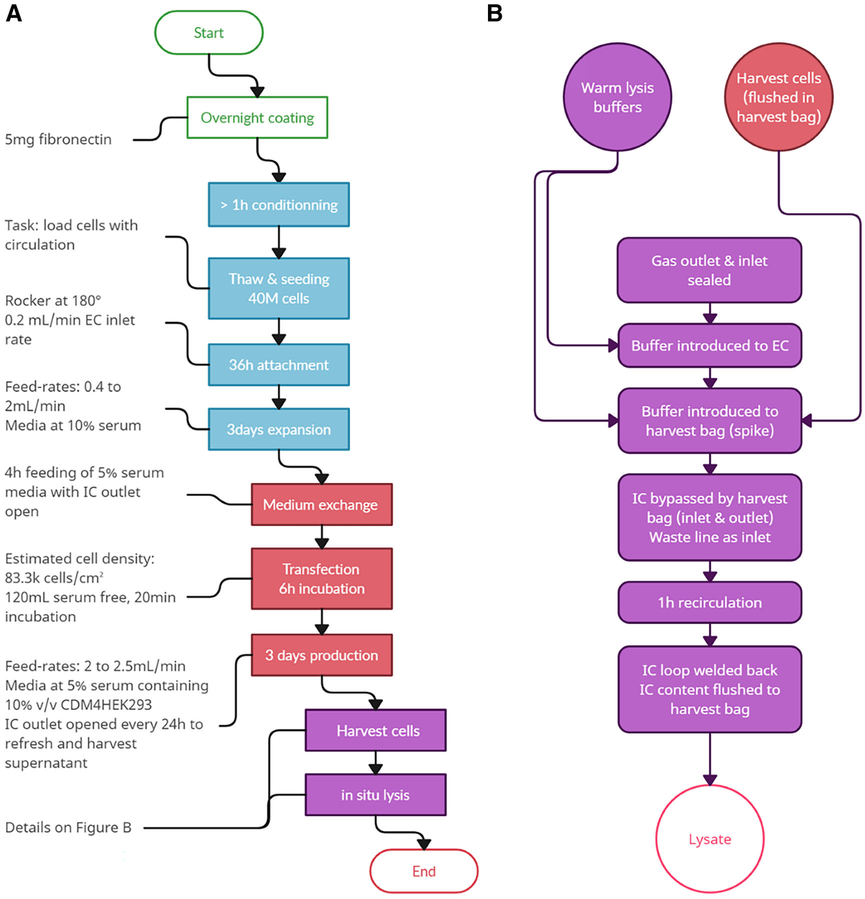

Figure 2. Quantum process flowchart

(A) An overview of the steps from cell thawing to *in situ* lysis.

(B) Detailed steps of harvest and lysis. All steps were achieved using predefined custom tasks that were used for all engineering runs to maintain consistency.

remained stable at 0.5 mM from day 4 for the remainder of production. All the runs demonstrated comparable trends across all four metabolites (Figure 4B).

Engineering runs—AAV2 production titers

Viral genomes titer (vg) was calculated for the crude lysate by quantitative polymerase chain reaction (qPCR), and the vg/mL was used to calculate the total viral yield. The Quantum harvest volume was approximately 1.2 L containing a mean (n = 3) of 4.92 × 10^{14} vp (4.20 × 10^{11} ± 0.61 × 10^{11} vp/mL) and 6.81 × 10^{13} vg (5.81 × 10^{10} ± 1.40 × 10^{10} vg/mL), from approximately 5.0 × 10^{9} lysed cells, demonstrating substantial and consistent productivity from the system (Figure 5). This calculates to an average full particle ratio of 14%.

Engineering runs—lysate purification

Of the three engineering runs performed, runs 2 and 3 were processed using a representative downstream process (DSP) workflow. The harvest material from the first run was used to confirm DSP parameter ranges and experimental configuration. Previous DSP optimization was conducted at small scale (<100 mL feed material), so process parameters required confirmation at increased scale prior to adoption in engineering runs 2 and 3. Primary and secondary clarification unit operations are necessary to prepare the crude lysate feed stream by removing colloids, ensuring the efficiency and performance of the subsequent membranes and chromatographic capture resin, thus providing and controlling the biochemical properties of the AAV2 for optimal drug substance production.

Primary clarification of run 2 and run 3 lysate material demonstrated comparable operation. A steep increase in inlet pressure to 25—30 psig was observed, following the processing of approximately 440 mL (10 mL/cm^2). The pressure profile plateaued during the processing of the remaining volume (Figure 6A). As a key indicator of performance, this operating pressure range was below that of the critical 80% P_{max} at 35 psig, indicating adequate processibility capacity for the feed stream.

Secondary clarification in series demonstrated a steady inlet pressure increase, not exceeding 5 psig for either run. This demonstrated effective large colloid removal during primary clarification, with secondary clarification removing any remaining high-molecular-weight particles (Figure 6A). Turbidity measurements, often used to assess cell density, particle concentration, and size distribution in the crude harvest, supported the effectiveness of this clarification process.[19] Depth filtration of the complex lysate (Figure 6A) resulted in a 97.0% reduction in lysate turbidity (Figure 6A; measured using recorded change in nephelometric turbidity unit [NTU]), yielding 90.0% vp recovery (+/− 20% [range of 69%–104%], Figure 6B), demonstrating effective clarification operation using Quantum produced lysate material.

Ultrafiltration/diafiltration (UF/DF) operation using tangential flow filtration (TFF) was performed to reduce the volumetric batch size, washout solvent/detergent and low-molecular-weight particles, and help stabilize the AAV2 drug substance for optimal chromatography capture. Concentration and buffer exchange via TFF was conducted, and performance, as outlined in Figure 6C, demonstrates a steady decrease in flux (Jp, filtrate flux rate) from 60 to 50 and 70 to 59 LMH (liter per meter square per hour), respectively, following the 5-fold volume reduction procedure (measured in volumetric reduction factor, VRF). The resulting unit operation yield was 90% vp (+/− 20%), as highlighted in Figure 6D. This operation also led to a 20% mean reduction in HCP during run 2 and a 40% mean

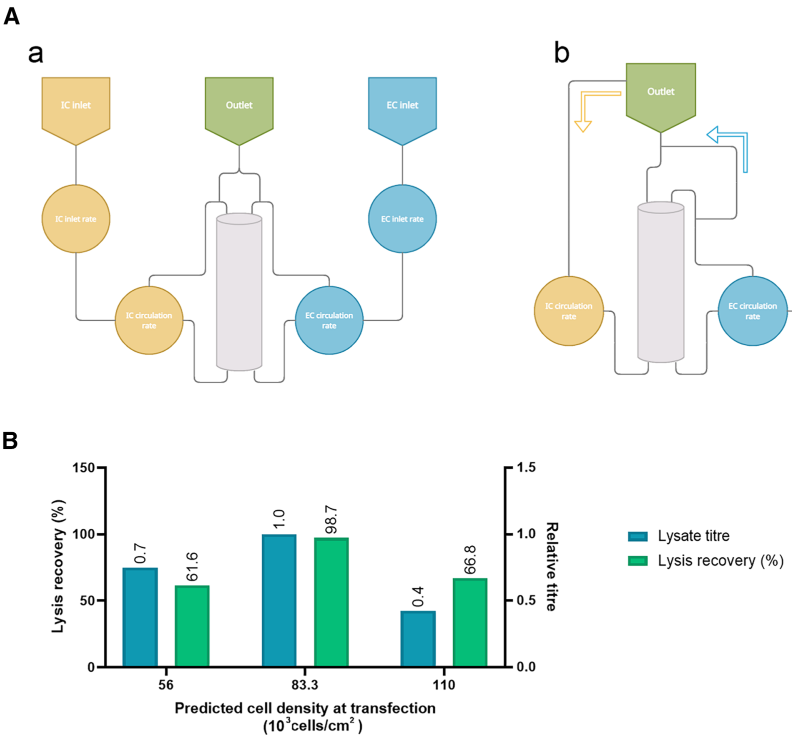

Figure 3. Development of the *in situ* lysis step

(A) A simplified fluid diagram of the Quantum bioreactor during production (left schematic [a]) and *in situ* lysis (right schematic [b]).

(B) Lysis performance obtained during process optimization for both titer and step recovery, with lysate titer reported relative to the best performing condition (83.3 × 10^3/cm^2 at transfection).

reduction in HCP for run 3, despite differing starting levels of HCP detected in the lysate (1.43 × 10^8 ng average and 2.27 × 10^8 ng average, respectively; Figure 6E). The differences in HCP and DNA quantities in run 2 and run 3 likely resulted from the different cell numbers produced in each run, coupled with variable impact of the lysis step. Total DNA clearance of 89% was demonstrated (from 1.8 × 10^7 ng to 2.0 × 10^6 ng and from 2.9 × 10^7 ng to 3.4 × 10^6 ng in runs 2 and 3, respectively) within both runs, despite slightly elevated starting levels in run 3 compared with run 2 (Figure 6F).

Cost of goods model

A cost of goods (CoG) model was developed internally, using Microsoft Excel. CoG metrics were used to compare the Quantum bioreactor, HYPERStack, and CS10 systems for a fixed facility (60 m^2) (Table 1; Figure 7). Outputs of the model were cost per batch (materials and facilities), process risks (open steps), and facility usage. The CoG modeling was updated after the execution of the three engineering runs to integrate experimental data into the model, whereas for the HYPERStack and CS10 system, data from our internal 2D production platform were used. The model output shows the performance of the three systems across an output range of 1 × 10^{14} and 1 × 10^{15} as would be required for a typical translational study. Across the three production scenarios, the cost per batch for HYPERStack was between 1.1- and 2-fold higher compared to the Quantum bioreactor (Figure 7A). The CS10 system was 11.4- to 20.7-fold more expensive than the Quantum system, decreasing with higher yield (Figure 7A). Both the HYPERStack and CS10 systems had >40-fold more open steps than the Quantum bioreactor (Figure 7B). The Quantum bioreactor reduced production time by 2- to 3.6-fold and 1.8- to 7.5-fold compared to the HYPERStack and CS10 systems, respectively (Figure 7C).

DISCUSSION

AAVs are recognized as a leading delivery vector for *in vivo* gene therapy, necessitating robust and economical manufacturing methods to produce clinical-grade material efficiently.[27] Current methodologies for producing small to medium yields of AAV predominantly involve growing insect or mammalian cells in adherent or suspension flasks under tissue culture conditions.[28] Recent clinical trials have illustrated the need for improved production methods capable of producing AAV.[8,27] Traditional, adherent methods, such as HYPERStack and CS10, rely on flask based techniques that are inefficient, requiring significant materials, facility footprint, and personnel time.[13,27,28] Additionally, the risk of contamination and batch-to-batch variability inherent with operating the HYPERStack or CS10 production systems makes them suboptimal for AAV production.[13–15]

Here, we report the development of a protocol for producing high-quality AAV material at yields appropriate for both translational research and clinical phase production. The workflow produced an AAV2 titer using the Quantum hollow-fiber bioreactor, incorporating a custom designed, *in situ* lysis step. This was complemented by a subsequent clarification, concentration, and buffer exchange workflow, demonstrating the processibility of the material liberated from Quantum. Thus, this approach facilitates the potential for automated, compact, and efficient production of AAV.

The semiautomated, sealed design of the bioreactor circumvents the need for manual steps such as exchanging media, cell passaging, and cell harvest, thereby demonstrating its suitability for the efficient production of AAV in fundamental translational research.[15] While the Quantum bioreactor operates as a closed system, pre- and post-Quantum processing techniques, such as reagent preparation and bag filling, are necessary to ensure end-to-end sterility throughout the AAV manufacturing process. One limitation of the system is its lack of sensing technology, requiring external analysis to be conducted to understand performance. To address this issue, the authors successfully integrated flow-through sensors for pH

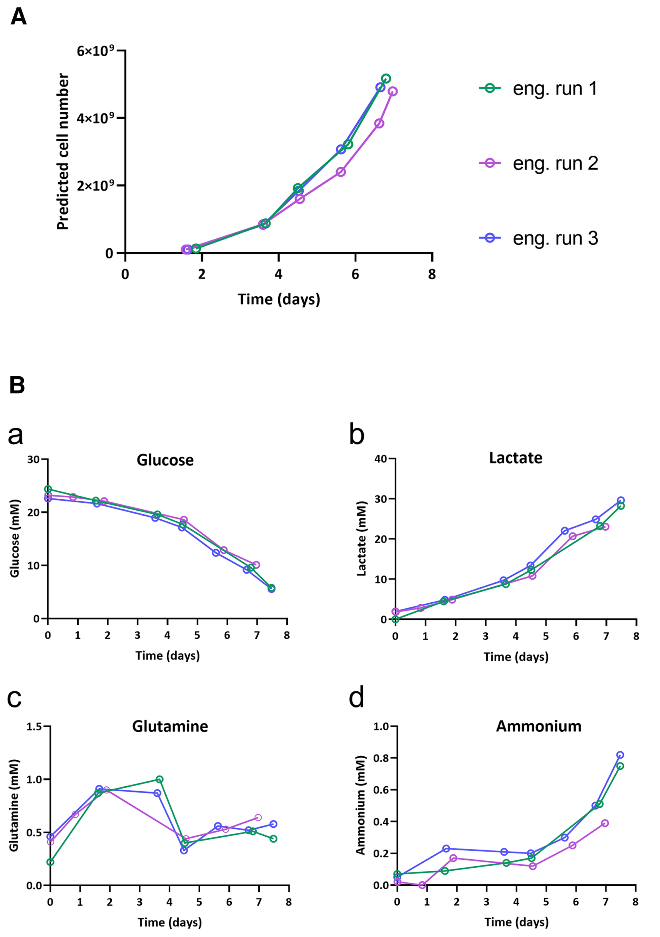

Figure 4. Engineering runs cell growth and metabolites

(A) Cell growth was predicted by lactate consumption and perfusion flow rates. (B) Metabolite concentrations that were measured were (a) glucose, (b) lactate, (c) glutamine, and (d) ammonium. Concentrations were determined using the Quantum bioreactor with samples being freshly analyzed from the EC side. Note: metabolite analysis was unable to be performed on day 8 of the second engineering run.

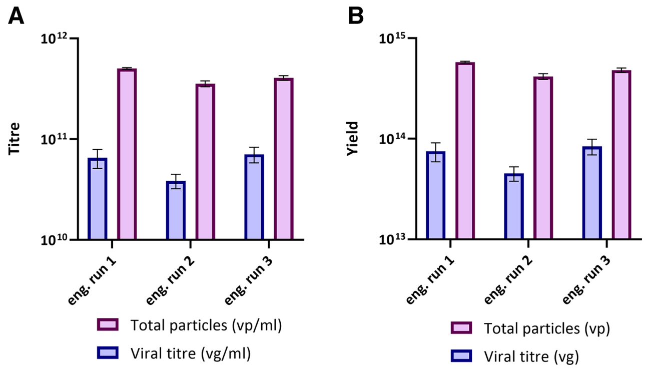

Figure 5. Engineering runs AAV2 production titers

(A) Titer of lysate per unit volume and (B) yield obtained from the harvest bag of the Quantum bioreactor. Total particles (ELISA) and viral titer (qPCR) were determined from lysate samples of harvest bag post-*in situ* lysis. qPCR samples were analyzed on the same plate. The bars represent the average titer (A) and yield (B) across the three engineering runs. The error bars represent the standard deviation for a given engineering run, calculated from nine measurements.

and oxygen available from PreSens (Germany) in other studies. New technology, such as the Maven from 908 devices, will also support in-line real-time sensing of analytes such as Glucose and Lactate. The study highlighted in this manuscript aimed to demonstrate comparable or improved AAV particle production using Quantum relative to traditional flask-based alternatives. Quantum production, on average, was equivalent to 3xCS10 and 0.5xHyperStack36 (based on theoretical values for flask production that are likely overestimated due to non-linear scaling).[16] Additionally, benefits regarding cost, open processing steps, and production time were also evaluated and are discussed below.

Due to the necessity for extensive and optimized purification of AAV, quantifying infectious units was not a primary objective of this study, given only clarification and buffer exchange was performed to represent the initial unit operations of a downstream workflow. However, additional capture and sample preparation were performed, and an infectious titer assay was conducted (using a previously reported method[29]) for material produced in engineering runs 2 and 3, confirming infectivity of a subset of particles produced (less than 0.1% of total vg—data not shown). Infectivity is specific to a wide range of variables, notably serotype, target tissue, and purity of vector preparation; therefore, it is recommended that a targeted infectivity assay that has utilized an adequate and well characterized purification workflow be employed to evaluate and quantify AAV produced using this method of production in future studies.

In recent years, new GMP-compliant transfection reagents, such as FectoVIR-AAV, have demonstrated 3-fold higher physical titers (both vg and vp) relative to PEI-pro, while maintaining similar cell viability (>80%).[30,31] Future work could explore incorporating these new transfection reagents in the workflow to maximize physical titers post-harvest. Alternative attachment substrates could also be investigated to further reduce the cost of materials, such as a substitute of fibronectin for vitronectin, as an example. In addition to new production reagents becoming available, other reagents used in this study have since been limited or controlled due to their environmental impact (e.g., Triton X-100). As such, an alternative lysis agent is required, such as Tween 20 or Tween 80.

Regarding downstream purification processes, a representative clarification workflow demonstrated that clarified AAV2 batches achieved 90.0% vp recovery and a 97.0% reduction in lysate turbidity, a common attribute linked to clarification. Subsequent concentrate and washing using TFF facilitated continuous, low-shear ultrafiltration/diafiltration of the clarified stream. High vector particle recovery was achieved (90%), as was significant clearance of HCP (up to 40%) and total DNA (89%). Impurity removal from lysate is a common bottleneck during clinical-scale AAV production,[32,33]

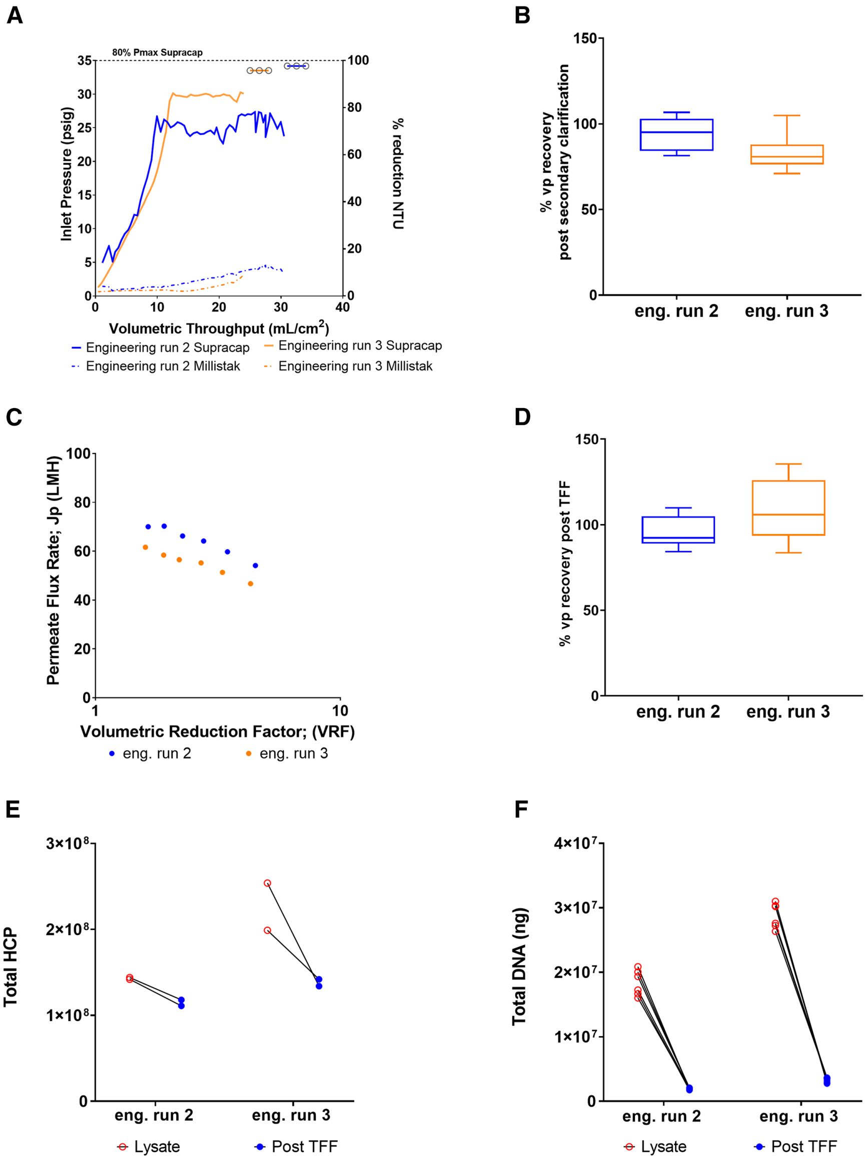

Figure 6. Engineering runs lysate purification

Parameters measured during primary and secondary clarification in series were (A) inlet pressure (psig) per volumetric throughput (mL/cm^2) and turbidity reduction, (B) total particle recovery as determined by vector particle ELISA. For concentration and buffer exchange via tangential flow filtration (TFF), the results shown are (C) permeate flux rate (plotted as Jp (LMH) per VRF), (D) total particle recovery as determined by vector particle ELISA, (E) total HCP reduction, and (F) reduction in residual DNA from engineering runs 2 and 3. Data in (B) and (D) are represented via standard box and whisker plots.

highlighting the suitability for translatable manufacturing of the lysate produced within the Quantum bioreactor and the corresponding downstream process reported.[32]

To substantiate the rationale for adopting the Quantum bioreactor approach over traditional flask-based methods, we developed a range of operational comparison models focusing on cost and risk analysis; cost per batch, open steps per batch, and time of (batch) production. Within a range of production between 1×10^{14} and 1×10^{15} viral genomes (vg), our analysis demonstrates a favorable productivity in terms of all three metrics measure, with greater impact versus both the CS10 unit and HYPERStack unit approach. These findings align with prior studies, the first of which achieved mesenchymal stem cell expansion in the Quantum bioreactor 9 days faster than flask-based approaches and the second demonstrating a 2-fold reduction in production costs when manufacturing 1.0×10^8 mesenchymal stem cells using Quantum versus traditional methods.[18,19]

In this analysis, the CoG model demonstrated that Quantum offered favorable output with respect to all three metrics. The advantage of this system is that it operates efficiently in a compact facility (60 m^2), requiring fewer operators and less laboratory space, as one skilled user can operate up to 10 Quantum bioreactors simultaneously.[34] The Quantum bioreactor surface area is 2.1 m^2, which is the equivalent size to 3.3xCS10 and 1.17xHyperStack36. While the Quantum bioreactor maintains large surface areas for cell growth, savings can be made due to reduced procedure time, space, and personnel, which compensate for initial investment in the system. Fewer open steps also limit the potential costs of microbial contamination. In line with previous studies, accounting for such factors highlights the cost-effective benefits of selecting the Quantum bioreactor over traditional cell culture methods, especially in academic or smaller industrial laboratories. To confirm the CoG results obtained internally, there is a need to perform a similar analysis using established and standardized modeling software; one such example is Biosolve from Biopharm.

In conclusion, a novel approach for producing a clinically relevant AAV titer is presented, complemented by an initial risk and CoG assessment. The workflow used a small-footprint hollow-fiber bioreactor to generate a closed and semi-automated production platform, which can process a large quantity of biomass feed stream to deliver highly purified AAV2 particles. Compared to existing small- to medium-scale methods for vector production, the method presented in this study produces a comparable or higher AAV2 titer, accompanied by reduced costs, number of open steps, and production time required. These findings provide preliminary evidence that

Table 1. Cost of goods model assumptions

**Experimental**
• Quality control costs were calculated per sample and includes qPCR and ELISA analysis<br>• Quality control costs assumed each plate is full to share the analytical costs across samples, taking advantages of the volume of analytics required<br>• Quantum yield was on average a total of 6.81×10^{13} vg across three engineering runs<br>• CS10 (Corning CellSTACK 10-layer) and HYPERStack total yields were extrapolated from internal production titers at an average of 3.50×10^{10} vg. mL^{-1} in 2D flasks with AAV2 production process, obtaining 2.45×10^{13} vg for CS10 and 1.26×10^{14} vg for HYPERStack; this assumes linear scale up, which is commonly agreed to be unlikely, thus giving an advantage to plastic based systems in this model<br>• DSP (downstream process) recovery was set at 30% from harvested lysate through to drug product formulation, inclusive of the compounded losses associated with all required unit operations, e.g., for clarification, TFF, capture, polishing, and formulation stages
**Operational**
• Quantum operation was set to 6.0 m^2 of bench space for 10 systems, requiring two operators and one MSC (microbial safety cabinet) with an air-grade C<br>• Incubator space (168 L) was assumed as one CS10 or HYPERStack® unit per incubator<br>• For a 6-unit batch of either CS10 or HYPERStack® per MSC, it was assumed five operators would be required during production, operating for a maximum of three MSCs within a Grade B environment<br>• CS10 and HYPERStack® manipulations were fixed at six units per every MSC<br>• The scheduling section assumed two GMP (Good Manufacturing Practice) operators per MSC<br>Number of doses produced and the capacity of patients to treat were also determined (data not shown) and are dependent of production outputs<br>Each upstream batch was split into several productions if the capacity of the facility (60.0 m^2) was reached<br>The size of the required equipment was included, and a 2.0 m^2 space was set for each operator to move around

Quantum may be an efficient and cost-effective system for facilities with limited resources, such as hospital production and academic research facilities.

MATERIALS AND METHODS

Cost of goods assumptions

Three production systems, namely the Quantum, CS10, and Corning 36-layer HYPERStack 36, were studied through a CoG model, an internally developed tool using Microsoft Excel software. The tool developed considers costs of raw materials as well as costs of equipment, operator salaries, and facility operating costs. A process is defined within the tool by establishing a production schedule, within which every day will consist of process steps that are attributed with their own raw materials, operators, and time requirements. Initial parameters are then required, for example size of the facility, product quantity desired, upstream yields, and downstream recoveries. Outputs are then generated—for this study, the cost of a batch that will generate the desired quantity of product, as well as the number of open steps and time to produce this batch. The model assumed that both experimental and operational parameter assumptions were consistent across all systems (Table 1).

Cell culture

Production of AAV2 vectors was performed in a 2D cell culture environment using Human Embryonic Kidney 293T (HEK293T) cells. HEK293T cells were used under a non-exclusive licence from Dr Frank L. Graham (AdVec Inc, Ontario, Canada). The cells were cultured in Dulbecco's Modified Eagle's Medium (DMEM) (Thermo Fisher Scientific, cat. 115744862) supplemented with 10% FBS and 2mM of GlutaMAX (Gibco, cat. 35050061). A high-density cell bank was generated by cryopreserving 10×10^6 cells per vial into 1 mL of CryoStor CS10 Cell Freezing Medium (StemCell Technologies, cat. 07930).

Historical AAV production

Historically, prior to transfection, cell culture media was replaced by fresh serum free media consisting of DMEM supplemented with 2mM of L-Glutamine (Gibco, cat. 11539876). Triple plasmid

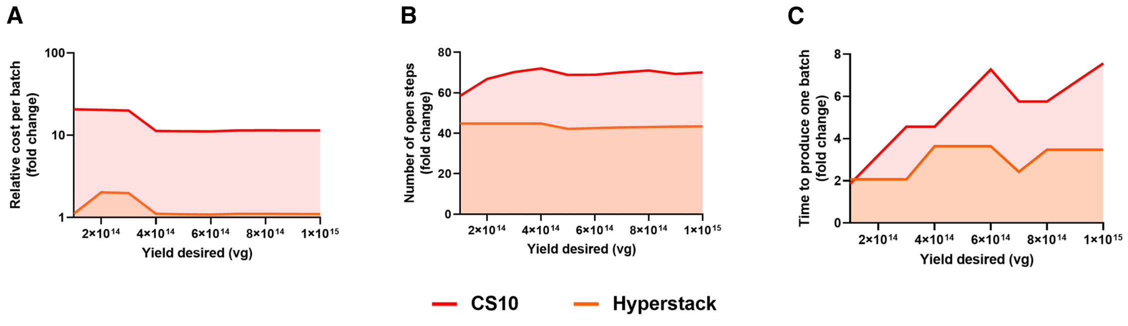

Figure 7. Cost and risk analysis

CoG metrics used to determine process desirability are shown in Table 1 for a fixed facility (60 m^2), including (A) costs (materials, facilities, etc.), (B) process risks (open steps), and (C) facility usage; log10 scale is used across all figures. All metrics in this figure are shown in fold-changes relative to the Quantum process.

transfection of HEK293T cells was performed using a helper plasmid (E2A, E4, and VA), a GFP (green fluorescent protein) reporter gene plasmid, and a rep and cap plasmid encoding the AAV2 particle, in a 1:1:1 M ratio, respectively. The total amount of DNA used was 0.143 μg per cm^2. The PEIPro (Polyplus, cat. 115-100) transfection reagent was used (reagent to DNA ratio of 3:1). Both transfection reagent and DNA were diluted into OptiMEM serum-free medium (5% of final volume each) before being combined and incubated for 20 min, then added to the cells. Sixteen hours post-transfection, cell media was replaced by fresh DMEM media supplemented with 5% FBS and 2 mM of L-Glutamine. Two days after transfection, cells were detached using TrypLE (Thermo Fisher Scientific, cat. 12604054), then centrifuged at 2,500 x g for 10 min at 4°C. The supernatant was then collected, and cell pellet was lysed using a buffer constituted of 50Mm Tris pH 8, 150mM of NaCl, and 2mM of $MgCl_2$ and four sequential freeze-thaw cycles. Finally, the resulting lysed cells were treated with 50 units per mL of Benzonase, then centrifuged at 2,500 x g for 10 min at 4°C to obtain the clarified lysate.

Quantum bioreactor: Tasks

Detailed protocol used to perform those runs are available in the supplemental material, detailing the sequence of tasks and their parameters that need to run by the system. Each task can be started from the Quantum software, after adjusting settings of the task according to details in Tables S1–S6. If there is a custom task grouping, the custom tasks need to be input into the Quantum bioreactor systems prior the run. When a custom task is saved, it becomes available from the Quantum software like any other task. Each task is defined by a list of settings that are as follows. IC/EC inlet is the line used to feed the IC/EC compartment. IC/EC inlet rate is the feeding rate for the IC/EC compartment in mL/min. IC/EC circulation rate is the internal recirculation rate of the IC/EC compartment in mL/min; a negative value will be a counterflow recirculation. Outlet is the destination of outflow from the system, Harvest will be the harvest bag (1 L) attached onto the system, and IC/EC outlet will be a waste bag. Selecting Harvest or IC as an outlet will allow harvest or media replacement of the IC compartment, which introduces a risk of losing cells. Selecting EC as an outlet will remove media from the EC compartment with no impact on the cells. The rocker motion will be stationary or in movement. Its position is given by its angle (°), and if in motion two angles are given as a start and an end position with a waiting time in seconds between the two movement. The stop condition will be the condition to be met for the task to stop or to go to the next task if there is one; the condition can for example be a certain amount of time, a volume to be processed, or simply a manual intervention of the user.

Engineering runs, Quantum bioreactor—cell expansion

Figure 2 presents an overview of the steps needed for the three engineering runs. The Quantum bioreactor has two main fluid circuits, one primarily for cell culture (intra-capillary [IC] loop) and another for gas exchange (extra-capillary [EC] loop). Both can be controlled independently for feeding and waste removal. Briefly, to receive adherent cells, the bioreactor was prepared with an attachment substrate, in this case 5 mg of fibronectin (Sigma-Aldrich, cat. F0895-5MG). Following conditioning, a high-density cell bank was thawed and diluted in fresh media to obtain a total of 40.0×10^6 viable cells that were seeded onto the bioreactor via the IC loop and attached for 36 h. The cells were then expanded for 3 days using DMEM supplemented with 10.0% FBS and 2 mM of GlutaMAX.

Cell metabolites (glucose, lactate, glutamine, and ammonium) were measured daily on the BioProfile FLEX2 metabolites analyzer (Nova Biomedical, cat. 57528), with lactate measurements used to predict cell growth.

Engineering runs, Quantum bioreactor—cell transfection

A slow media exchange was performed by opening the IC outlet for 4 h to reduce serum concentration to 5.0%. Triple plasmid transfection of HEK293T cells was performed using a helper plasmid (E2A, E4, and VA), a GFP (green fluorescent protein) reporter gene plasmid, and a rep and cap plasmid encoding the AAV2 particle, in a 1:2:2 M ratio, respectively. The total amount of DNA used was 1.5 μg per million predicted cells. The PEIPro transfection reagent was used with a reagent to DNA ratio of 2:1. Both transfection reagent and DNA were diluted into OptiMEM serum-free medium (60 mL final volume each) before being combined and incubated for 20 min.

The final resulting 120 mL was then added directly to the bioreactor, which was then left to incubate for 6 h. After incubation, media feeding is automatically restarted. During the 3 days of production, cells were fed with media containing 5.0% serum, 2mM of GlutaMAX, and 10.0% v/v CDM4HEK293 media (Cytiva, cat. SH30858.02).[35]

Engineering runs, Quantum bioreactor—*in situ* lysis steps

Simplified fluid diagrams of the Quantum bioreactor during production and *in situ* lysis are presented in Figure 3A. Briefly, the Quantum bioreactor consists of a hollow-fiber cartridge with IC and EC loops that can be used to load cells and add or remove media, reagents, and waste (Figure 3A, schematic a).

Cells were detached with TrypLE and transferred to the harvest bag. The gas inlet and outlet were manually sealed with the Terumo handheld tube sealer to prevent leaks during fluid recirculation when performing *in situ* lysis. The 1.5 L EC lysis buffer was then introduced to the EC volume, containing 3 mL of magnesium chloride and 45 mL of sodium chloride. The system was paused, the harvest bag taken into a microbial safety cabinet to be spiked (luer spike), and the *in situ* lysis buffer containing 2 mL of magnesium chloride, 30 mL of sodium chloride, 20 mL of Triton X-100, and 134 μL of benzonase (Merck Millipore, cat. E1014-5KU) introduced via a luer syringe (VWR, cat. 613–2053). The syringe was then disconnected and replaced with a “three-way connecter” (manufactured by cutting one end of the other two tubing pieces and inserted into the other two ends of the “T” connecter to create a bypass) (Figure S2).

The IC loop was sealed and cut open with the Terumo handheld tube sealer. One end was welded to one of the previously made available ends on the three-way connecter, and the other end was welded to the tube originally on the harvest bag. The last free available ends on the three-way connecter (V) were then welded to the waste line (Figure 3A, schematic b). The harvest bag was then placed outside of the Quantum bioreactor (on the bag holder), and the process was resumed to complete the lysis procedure. When the procedure was complete (approximately 1 h), the harvest bag was disconnected with the Terumo handheld tube sealer.

Engineering runs 2 and 3, primary and secondary clarification

Primary and secondary clarification unit operations were necessary to prepare the crude lysate feed stream for further purification unit operations. To remove both large product and process-related impurities, clarification of the lysate was performed using primary and secondary depth filtration in series. The P_{max} approach was used to process the crude lysate at a constant flow rate, while the feed inlet pressure was monitored. The primary depth filter technology used for clarification was two adjoined units of SUPRACap HP PDH4 (Pall Corporation, cat. SC050PDH4), offering high permeability and a suitable retention rating of 0.5–15 μm. The combined filter surface area was 44 cm^2. Connected in series were two adjoined units of the secondary depth filter technology, the Millistak C0HC (Merck Millistak, cat. MC0HC027H1). This technology offered a pore size rating of 0.5–9.0 μm, with a combined filter surface area of 46 cm^2. The connectivity facilitated a single step clarification process, to aid scalability.

Turbidity

Sample turbidity was measured using the Orion AQ4500 Turbidimeter (Thermo Scientific) and was monitored using the infrared mode as the turbidity measurement.

Engineering runs 2 and 3, tangential flow filtration

Clarified AAV2 vectors were concentrated via ultrafiltration (UF) and buffer exchanged via diafiltration (DF) using the TFF module Pellicon 2 Biomax C-100 (Merck Pellicon, cat. P2B100C05). The clarified filtrate was directly connected to the TFF processing loop (1.2 L), concentrated five times, and diafiltered into a Tris-ethylenediaminetetraacetic acid (TE) buffer. A cross-flux rate of 7 LMM was used to process the clarified material, with a fixed transmembrane pressure (TMP) of 10 psig during both runs. A concentration/diafiltration/concentration (C/D/C) approach was used for UF followed by a C/D approach for DF. The product was collected for a final flush with DF buffer at twice the hold-up volume.

Viral genomes quantification—qPCR

The viral genomes (vg) titer was calculated on the Quantstudio 7 (Thermo Fisher, cat. 4485701). Briefly, AAV containing samples were treated with DNase I (Sigma, cat. D5307) to remove residual DNA that was not encapsulated within the viral particles. Nuclease-treated samples were diluted using a 5-point dilution series followed by temperature lysis of the AAV particles. The extracted genomic DNA was further diluted prior to PCR and detection performed using primers and probes targeting the ITRs.[36] Calculated titer is reported as vector genomes per milliliter (vg/mL).

Viral particle quantification—ELISA

Viral particles (vp) were measured using an AAV2 enzyme-linked immunosorbent assay (ELISA). The assay was performed according to the manufacturer's instructions (ProGen, cat. PRATV). Samples were diluted to align with the linear range of the assay, in line with the viral genomes data obtained from the qPCR analysis coupled with an estimation regarding the full to empty ratio achieved (informed by previous in-house testing). Samples were quantified using a TECAN Infinite M1000 plate reader (96-well format) (Tecan, cat. Infinite M1000 PRO). Calculated particle number is reported as vector particle per milliliter (vp/mL).

Host cell proteins enzyme-linked immunosorbent assay

Host cell protein (HCP) levels were determined using a HEK293T HCP ELISA kit (Cygnus Technologies, cat. F650S). The assay was performed according to the manufacturer's instructions and utilized the TECAN Infinite M1000 plate reader (96-well format) (Tecan, cat. Infinite M1000 PRO). Samples were diluted within the range of 1:20 to 1:80, to align with the linear range of the assay.

PicoGreen assay

Residual dsDNA was quantified using the QuantiT PicoGreen dsDNA Assay Kit (Molecular Probes, Invitrogen, cat. P11496) and utilized the TECAN Infinite M1000 plate reader (96-well format) (Tecan, cat. Infinite M1000 PRO). The assay was performed according to the manufacturer's instructions.

DATA AVAILABILITY

Data supporting this study are included within the article and/or supporting materials (can be provided by CGTC upon request).

ACKNOWLEDGMENTS

This work was funded as part of a wider collaboration with Professor Farzin Farzaneh's group at Kings College London and with Guy's and St Thomas' NHS Foundation Trust. The authors wish to acknowledge the support of Andrea Sirianni in preparation of the manuscript and Stuart Gibb of Terumo BCT for technical discussion.

Funding source: this work was performed by the Cell and Gene Therapy Catapult with funding from Innovate UK.

AUTHOR CONTRIBUTIONS

All authors participated in the conception, design, and implementation of the study. All authors were involved in the interpretation of analyzed data and the decision to submit for publication. Medical writing support was provided by Bham Pharma Ltd, UK.

DECLARATION OF INTERESTS

The authors have no conflicts of interests to declare.

SUPPLEMENTAL INFORMATION

Supplemental information can be found online at https://doi.org/10.1016/j.omtm.2025.101496.

REFERENCES

1. Pan, X., Veroniaina, H., Su, N., Sha, K., Jiang, F., Wu, Z., and Qi, X. (2021). Applications and developments of gene therapy drug delivery systems for genetic diseases. Asian J. Pharm. Sci. *16*, 687–703.
2. Wang, J.H., Gessler, D.J., Zhan, W., Gallagher, T.L., and Gao, G. (2024). Adeno-associated virus as a delivery vector for gene therapy of human diseases. Signal Transduct. Target. Ther. *9*, 78.
3. Naso, M.F., Tomkowicz, B., Perry, W.L., and Strohl, W.R. (2017). Adeno-Associated Virus (AAV) as a Vector for Gene Therapy. BioDrugs *31*, 317–334.
4. Becker, J., Fakhiri, J., and Grimm, D. (2022). Fantastic AAV Gene Therapy Vectors and How to Find Them—Random Diversification, Rational Design and Machine Learning. Pathogens *11*, 756.
5. Gaudet, D., Méthot, J., Déry, S., Brisson, D., Essiembre, C., Tremblay, G., Tremblay, K., de Wal, J., Twisk, J., van den Bulk, N., et al. (2013). Efficacy and long-term safety of alipogene tiparvovec (AAV1-LPLS447X) gene therapy for lipoprotein lipase deficiency: an open label trial. Gene Ther. *20*, 361–369.
6. Maguire, A.M., Russel, S., Wellman, J.A., Chung, D.C., Yu, Z.F., Tillman, A., Wittes, J., Pappas, J., Elci, O., Marshall, K.A., et al. (2019). Efficacy, Safety, and Durability of Voretigene Neparvovec-rzyl in RPE65 Mutation–Associated Inherited Retinal Dystrophy: Results of Phase 1 and 3 Trials. Ophthalmology Sep *126*, 1273–1285.
7. Ramamurthy, R.M., Atala, A., Porada, C.D., and Almeida-Porada, G. (2022). Organoids and microphysiological systems: Promising models for accelerating AAV gene therapy studies. Front. Immunol. *13*, 1011143.
8. Elangkovan, N., and Dickson, G. (2021). Gene Therapy for Duchenne Muscular Dystrophy. J. Neuromuscul. Dis. *8*, S303–S316.
9. Wright, J.F. (2008). Manufacturing and characterizing AAV-based vectors for use in clinical studies. Gene Ther. *15*, 840–848.
10. Graham, F.L., Smiley, J., Russel, W.C., and Nairn, R. (1977). Characteristics of a Human Cell Line Transformed by DNA from Human Adenovirus Type 5. J. Gen. Virol. *36*, 59–74.
11. Ogston, P., Raj, K., and Beard, P. (2000). Productive replication of adeno-associated virus can occur in human papillomavirus type 16 (HPV-16) episome-containing keratinocytes and is augmented by the HPV-16 E2 protein. J. Virol. *74*, 3494–3504.
12. De, B.P., Cram, S., Lee, H., Rosenberg, J.B., Sondhi, D., Crystal, R.G., and Kaminsky, S.M. (2023). Assessment of Residual Full-Length SV40 Large T Antigen in Clinical-Grade Adeno-Associated Virus Vectors Produced in 293T Cells. Hum. Gene Ther. *34*, 697–704.
13. Masri, F., Cheeseman, E., and Ansorge, S. (2019). Viral vector manufacturing: how to address current and future demands? Cell Gene Ther. Insights *5*, 949–970.
14. Titus, K., Klimovich, V., Rothenberg, M., Pardo, P., Tanner, A., and Martin, G. (2010). Closed system cell culture protocol using HYPERStack® vessels with gas permeable material technology. J. Vis. Exp. 2499.
15. Comisel, R.-M., Kara, B., Fiesser, F.H., and Farid, S.S. (2021). Lentiviral vector bioprocess economics for cell and gene therapy commercialization. Biochem. Eng. J. *167*, 107868.
16. Cameau, E., Pedregal, A., and Glover, C. (2019). Cost modelling comparison of adherent multi-trays with suspension and fixed-bed bioreactors for the manufacturing of gene therapy products. Cell Gene Ther. Insights *5*, 1663–1674.
17. Hulme, C.H., Mennan, C., McCarthy, H.S., Davies, R., Lan, T., Rix, L., Perry, J., and Wright, K. (2023). A comprehensive review of quantum bioreactor cell manufacture: Research and clinical applications. Cytopathology (Oxf.) *25*, 1017–1026.
18. Rojewski, M.T., Fekete, N., Baila, S., Nguyen, K., Fürst, D., Antwiler, D., Dausend, J., Kreja, L., Ignatius, A., Sensebé, L., and Schrezenmeier, H. (2013). GMP-Compliant Isolation and Expansion of Bone Marrow-Derived MSCs in the Closed, Automated Device Quantum Cell Expansion System. Cell Transplant. *22*, 1981–2000.
19. Russell, A.L., Lefavor, R.C., and Zubair, A.C. (2018). Characterization and cost-benefit analysis of automated bioreactor-expanded mesenchymal stem cells for clinical applications. Transfusion *58*, 2374–2382.
20. Bellio, M.A., Kanashiro-Takeuchi, R.M., Takeuchi, L., Kulandavelu, S., Lee, Y.-S., Balkan, W., Young, K.C., Hare, J.M., and Khan, A. (2022). Systemic delivery of large-scale manufactured Wharton's Jelly mesenchymal stem cell-derived extracellular vesicles improves cardiac function after myocardial infarction. J. Cardiovasc. Aging *2*, 9.
21. Vymetalova, L., Kucirkova, T., Knopfova, L., Pospisilova, V., Kasko, T., Lejdarova, H., Makaturova, E., Kuglik, P., Oralova, V., Matalova, E., et al. (2020). Large-Scale Automated Hollow-Fiber Bioreactor Expansion of Umbilical Cord-Derived Human Mesenchymal Stromal Cells for Neurological Disorders. Neurochem. Res. *45*, 204–214.
22. Hanley, P.J., Mei, Z., Durett, A.G., Cabreira-Hansen, M.d.G., Klis, M., Li, W., Zhao, Y., Yang, B., Parsha, K., Mir, O., et al. (2014). Efficient Manufacturing of Therapeutic Mesenchymal Stromal Cells Using the Quantum Cell Expansion System. Cytopathology (Oxf.) *16*, 1048–1058.
23. Sheu, J., Beltzer, J., Fury, B., Wilczek, K., Tobin, S., Falconer, D., Nolta, J., and Bauer, G. (2015). Large-scale production of lentiviral vector in a closed system hollow fiber bioreactor. Mol. Ther. Methods Clin. Dev. *2*, 15020.
24. Coeshott, C., Vang, B., Jones, M., and Nankervis, B. (2019). Large-scale expansion and characterization of CD3+ T-cells in the Quantum® Cell Expansion System. J. Transl. Med. *17*, 258.
25. Cunningham, A.W., Jones, M., Frank, N., Sethi, D., and Miller, M.M. (2022). Stem-like memory T cells are generated during hollow fiber perfusion-based expansion and enriched after cryopreservation in an automated modular cell therapy manufacturing process. Cytopathology (Oxf.) *24*, 1148–1157.
26. Vandenberghe, L.H., Xiao, R., Lock, M., Lin, J., Korn, M., and Wilson, J.M. (2010). Efficient serotype-dependent release of functional vector into the culture medium during adeno-associated virus manufacturing. Hum. Gene Ther. *21*, 1251–1257.
27. Li, C., and Samulski, R.J. (2020). Engineering adeno-associated virus vectors for gene therapy. Nat. Rev. Genet. *21*, 255–272.
28. Moleirinho, M.G., Silva, R.J.S., Alves, P.M., Carrondo, M.J.T., and Peixoto, C. (2020). Current challenges in biotherapeutic particles manufacturing. Expert Opin. Biol. Ther. *20*, 451–465.
29. American Type Culture Collection (ATCC). AAV2 RSS Infectious Titer Assays. Available at: https://www.atcc.org/-/media/product-assets/documents/protocols/virology/aav2-rss-infectious-titer-assays.pdf.
30. Hebben, M. (2018). Downstream bioprocessing of AAV vectors: industrial challenges & regulatory requirements. Cell Gene Ther. Insights *4*, 131–146.
31. Nyamay'antu, A., Hellal, M., Porte, M., and Erbacher, P. (2020). FectoVIR®-AAV: a giant step for AAV large scale manufacturing. Cell Gene Ther Insights *6*, 655–661.
32. Wang, D., Tai, P.W.L., and Gao, G. (2019). Adeno-associated virus vector as a platform for gene therapy delivery. Nat. Rev. Drug Discov. *18*, 358–378.
33. Chinnawar, R., and Marchand, N. (2022). Clarification of recombinant adeno-associated virus (rAAV) & lentivirus from adherent culture. Cell Gene Ther. Insights *08*, 483–493. Available from:. https://doi.org/10.18609/cgti.2022.070.
34. TerumoBCT (2025). QUANTUM® cell expansion system. Available at: https://www.terumobct.com/en/gl/products-services/cell-therapy-technologies/cell-therapy-technologies-products/quantum-flex.html.
35. Cytiva. (2025). Hyclone Media and Supplements CDM4HEK293. https://www.cytivalifesciences.com/en/us/shop/cell-culture-and-fermentation/media-and-feeds/specialty-media/hyclone-cdm4hek293-media-p-06207.
36. Aurnhammer, C., Haase, M., Muether, N., Hausl, M., Rauschhuber, C., Huber, I., Nitschko, H., Busch, U., Sing, A., Ehrhardt, A., and Baiker, A. (2012). Universal real-time PCR for the detection and quantification of adeno-associated virus serotype 2-derived inverted terminal repeat sequences. Hum. Gene Ther. Part B, Methods *23*, 18–28. https://doi.org/10.1089/hgtb.2011.034.

OMTM, Volume *33*

Supplemental information

The manufacture of AAV for gene therapy applications using a closed, semi-automated hollow-fiber bioreactor

Adrien Soula, Florian Leseigneur, Amna Anwar, Bilal Ozdoganoglu, Jagan Gurung, Hamza Bhatti, Juline Guenat, Quentin Bazot, Majahar Sayed, Carolina Pinto Ricardo, Lily Li, Katerina Farukshina, Tony Bou Kheir, Hadi Mirmalek-Sani, Gregory Berger, Julie Kerby, Jonathan Appleby, and Michael Delahaye

SUPPLEMENTAL MATERIAL

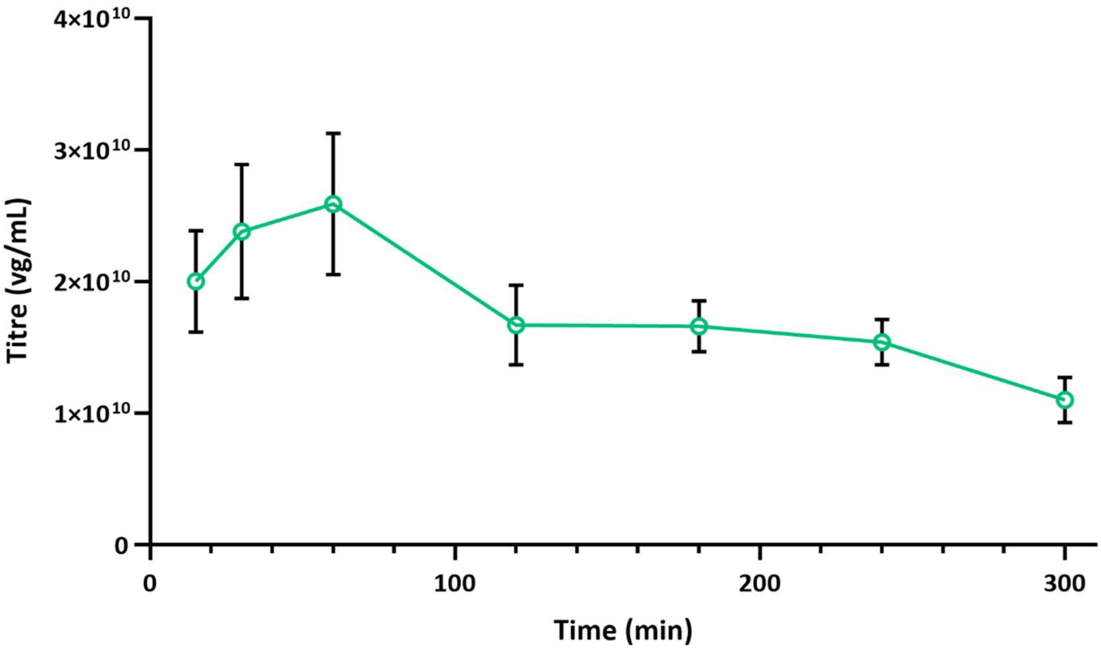

Figure S1: Quantum® lysis development. AAV2 viral genome titre resulting from time-course sampling, taken at 30-minute intervals across a 240-minute period to assess the impact of contact time on AAV2 liberation during the seventh Quantum® run. The error bars represent the standard deviation calculated from 9 measurements.

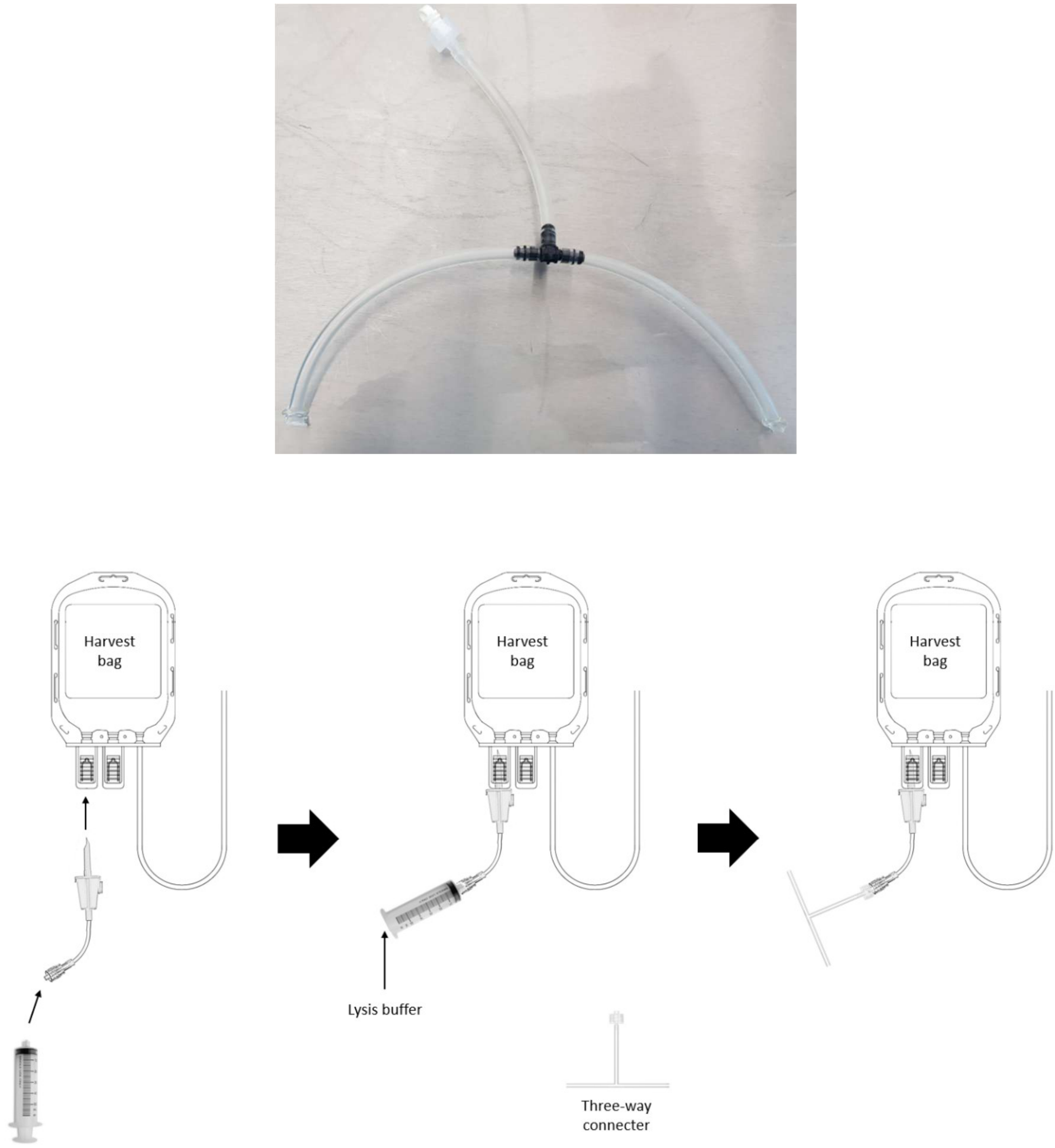

Figure S2: Fluid path adaptation. Use of three-way connecter to bypass the IC line, first a luer spike is used on the harvest bag, after lysis buffer addition, its luer end is used to seal the connection with the connecter.

Table S1: Quantum® Tasks settings for Day -1, set-up, priming and coating of the system. Reagent bag to contain fibronectin.

Day		*D-1*						
Task	Task Settings	Load Cell Expansion Set	Prime	Remove IC Air	Remove EC Air	Coat Bioreactor		
Custom Task Grouping		N/A	N/A	N/A	N/A	Step 1	Step 2	Step 3
		N/A	N/A	N/A	N/A	N/A	N/A	N/A
IC inlet		Default Settings	Default Settings	Inlet Source: Wash	Inlet Source: Wash	Reagent	Wash	None
IC inlet rate						10	10	0
IC circulation rate						100	100	20
EC inlet						None	None	Wash
EC inlet rate						0	0	0.1
EC circulation rate						30	30	30
Outlet						EC Outlet	EC Outlet	EC Outlet
Rocker Control						Stationary (0)	Stationary (0)	Stationary (0)
Stop condition						Empty Bag	IC Volume (22mL)	Manual
Time	Extra info	10 min	35 min	N/A	N/A	10 min	2 min	Overnight
Necessary Volume (estimation)		N/A	2L PBS	N/A	N/A	100 mL	22 mL	100 mL

Table S2: Tasks settings for Day 0, system conditioning and cell seeding. Cell bag to contain inoculum, media bags to contain media at 10% FBS.

**Day**		***D0***						
**Task**	**Task Settings**	**Inlet Line Wash-out**	**IC EC Washout**	**Condition Media**		**Load Cells with circulation**		
**Custom Task Grouping**		N/A	*Step 1*	*Step 2*	*Step 3*	Step 1	Step 2	Step 3
		N/A	CUSTOM TASK (1)			N/A	N/A	N/A
**IC inlet**		Source: Wash, Destination: Re-agent	IC Media	None	None	Cell	IC Media	IC Media
**IC inlet rate**			100	0	0	25	25	25
**IC circulation rate**			-17	100	100	139	139	-20
**EC inlet**			IC Media	IC Media	IC Media	None	None	None
**EC inlet rate**			148	0.1	0.1	0	0	0
**EC circulation rate**			-1.7	250	30	30	30	30
**Outlet**			IC & EC Outlet	EC Outlet	EC Outlet	EC Outlet	EC Outlet	EC Outlet
**Rocker Control**			In Motion	Stationary (0)	Stationary (0)	In Motion	In Motion	In Motion
			(-90, 180, 1)			(-90, 180, 1)	(-90, 180, 1)	(-90, 180, 1)
**Stop condition**			Exchange (2.5 IC, 2.5 EC)	Time (10 min)	Manual	Empty Bag	IC Volume (22 mL)	IC Volume (63 mL)
**Time**	**Extra info**	5 min	5 min	10 min	30-60 min	4 min	1 min	3 min
**Necessary Volume (estimation)**		N/A	800 mL	1 mL	<10 mL	100 mL	22 mL	63 mL

Table S3: Tasks settings for Day 0 to Day 5, cell attachment, cell feeding and media change prior to transfection. Media bags to contain media at 10% FBS, bag attached on IC media line for Step 8 of custom task 2 to contain media at 5% FBS.

**Day**		***D0 – D2***	***D2***	***D2 – D3***	***D3***	***D3 – D4***	***D4***	***D4 – D5***	***D5***
**Task**	**Task Settings**	**Attach Cells**	**Feed cells**						**Media change**
**Custom Task Grouping**		*Step 1*	*Step 2*	*Step 3*	*Step 4*	*Step 5*	*Step 6*	*Step 7*	*Step 8*
		CUSTOM TASK (2)							
**IC inlet**		None	IC media	IC media	IC media	EC media	EC media	EC media	IC media
**IC inlet rate**		0	0.2	0.4	0.8	0.8	1.2	1.6	2
**IC circulation rate**		0	-0.1	-0.1	-0.1	-0.1	-0.1	-0.1	0
**EC inlet**		IC media	IC media	IC media	IC media	EC media	EC media	EC media	None
**EC inlet rate**		0.2	0.2	0.4	0.4	0.4	0.4	0.4	0
**EC circulation rate**		30	50	100	100	100	100	100	100
**Outlet**		EC Outlet	EC Outlet	EC Outlet	EC Outlet	EC Outlet	EC Outlet	EC Outlet	Harvest
**Rocker Control**		Stationary (180)	Stationary (0)	Stationary (0)	Stationary (0)	Stationary (0)	Stationary (0)	Stationary (0)	Stationary (0)
**Stop condition**		Time (2,160 min)	Time (720 min)	Time (720 min)	Time (720 min)	Time (720 min)	Time (720 min)	Time (480 min)	Manual
**Time**	**Extra info**	36 h	12 h	12 h	12 h	12 h	12 h	8 h	4 h and up
**Necessary Volume (estima-tion)**		288 mL/day	576 mL/day	1,152 mL/day	1,728 mL/day	1,728 mL/day	2,304 mL/day	2,880 mL/day	500 mL

Table S4: Tasks settings for Day 5 to Day 7, transfection and cell feeding post-transfection with daily supernatant harvest. Reagent bag to contain transfection mix, media bags to contain media at 5% FBS, with 10% v/v of CDM4HEK293 from Step 5 of custom task 3.

Day		*D5*				*D5 – D6*	*D6*		*D6 – D7*
**Task**	**Task Settings**	**Add bag content**		**Incubate**		**Feed cells**	**Sup 24h**	**Feed cells**	
**Custom Task Group-ing**		*Step 1*	Step 2	Step 3	Step 4	Step 5	Step 6	Step 7	Step 8
		CUSTOM TASK (3)							
**IC inlet**		Reagent	IC media	None	None	EC media	EC media	EC media	IC media
**IC inlet rate**		10	10	0	0	0.2	2	0.2	0.2
**IC circulation rate**		50	50	-2	-2	-0.1	0	-0.1	-0.1
**EC inlet**		None	None	IC media	IC media	EC media	None	EC media	IC media
**EC inlet rate**		0	0	2	2	1.8	0	1.8	2.3
**EC circulation rate**		300	300	300	300	300	300	300	300
**Outlet**		EC outlet	EC outlet	EC Outlet	EC Outlet	EC Outlet	Harvest	EC Outlet	EC Outlet
**Rocker Control**		In Motion	In Motion	In Motion	Stationary (0)	Stationary (0)	Stationary (0)	Stationary (0)	Stationary (0)
		(-90, 180, 1)	(-90, 180, 1)	(0, 180, 1800)					
**Stop condition**		Empty bag	IC volume (47mL)	Time (120 min)	Time (240 min)	Time (960 min)	Time (120 min)	Time (720min)	Time (624 min)
**Time**	**Extra info**	10 min	2 min	2 h	4 h	16 h	2 h	12 h	10.4 h
**Necessary Volume (estimation)**		180 mL	47 mL	240 mL	480 mL	2,880 mL/day	240 mL	2,880 mL/day	3,600 mL/day

Table S5: Tasks settings for Day 7 to Day 8, cell feeding post-transfection with daily supernatant harvest and wash prior cell detachment. Media bags to contain 5% FBS and 10% v/v CDM4HEK293, wash bag to contain PBS.

**Day**		***D7***		***D7 – D8***	***D8***			
**Task**	**Task Settings**	**Sup 48h**	**Feed cells**		**Sup 72h**	**Wash**		
**Custom Task Grouping**		Step 9	Step 10	Step 11	Step 12	Step 13	Step 14	Step 15
		CUSTOM TASK (3)						
**IC inlet**		IC media	IC media	EC media	EC media	Wash	None	Wash
**IC inlet rate**		2.5	0.4	0.4	2.5	4	0	260
**IC circulation rate**		0	-0.1	-0.1	0	-2	0	-45
**EC inlet**		None	IC media	EC media	None	Wash	None	Wash
**EC inlet rate**		0	2.1	2.1	0	300	0	4
**EC circulation rate**		300	300	300	300	-3.5	0	2
**Outlet**		Harvest	EC Outlet	EC Outlet	Harvest	EC Outlet	EC Outlet	IC Outlet
**Rocker Control**		Stationary (0)	Stationary (0)	Stationary (0)	Stationary (0)	In Motion	Stationary (0)	In Motion
						(-90, 180, 1)		(-90, 180, 1)
**Stop condition**		Time (96 min)	Time (720 min)	Time (624 min)	Time (96 min)	EC Volume (500 mL)	Manual	IC Volume (234 mL)
**Time**	**Extra info**	1.6 h	12 h	10.4 h	1.6 h	5 min	N/A	5 min
**Necessary Volume (estimation)**		240 mL	3,600 mL/day	3,600 mL/day	240 mL	500 mL	N/A	240 mL

Table S6: Tasks settings for Day 8, cell detachment and *in-situ* lysis. Reagent bag to contain TrypLE, media bag to contain media at 10% FBS for custom task 4. Custom task 5 to be performed according to Figure 2, lines will be sealed, and bypass will be performed during the waiting task (step 2 of custom task 5). Lysate will be recovered in the harvest bag.

**Day**		***D8***								
**Task**	**Task Settings**	**Harvest cells**				***In-situ* Lysis**			**Rapid IC washout**	**Unload Cell Expansion Set**
**Custom Task Grouping**		Step 1	Step 2	Step 3	Step 4	Step1	Step 2	Step 3	N/A	N/A
		CUSTOM TASK (4)				CUSTOM TASK (5)			N/A	N/A
**IC inlet**		Reagent	Wash	None	IC media	Reagent	None	None	Reagent	Default Settings
**IC inlet rate**		30	30	0	400	40	0	o	260	
**IC circulation rate**		10	10	300	-70	-24	0	300	-45	
**EC inlet**		None	None	None	IC media	Reagent	None	Reagent	Reagent	
**EC inlet rate**		0	0	0	60	300	0	1.1	40	
**EC circulation rate**		30	30	30	30	-3.5	0	30	30	
**Outlet**		EC Outlet	EC Outlet	EC Outlet	Harvest	EC Outlet	EC Outlet	IC outlet	IC Outlet	
**Rocker Control**		In Motion	In Motion	In Motion	In Motion	In Motion	Stationary (0)	In Motion	In Motion	
		(-90, 180, 1)	(-90, 180, 1)	(-90, 180, 1)	(-90, 180, 1)	(-90, 180, 1)		(-90, 180, 1)	(-90, 180, 1)	
**Stop condition**		Empty Bag	IC Volume (22 mL)	Time (15 min)	IC Volume (378 mL)	EC Volume (500 mL)	Manual	60min	Exchange (1.3 IC, 0 EC)	
**Time**	**Extra info**	5 min	1 min	15 min	1 min	5 min	N/A	1 hour	5 min	5 min
**Necessary Volume (estimation)**		200 mL	22 mL	0mL	500 mL	800 mL	N/A	N/A	N/A	N/A

Table S7: Description of the first 4 Quantum® runs used to established cell expansion and vector production task settings.

Process step	1st run	2nd run	3rd run	4th run
**Seeding**	200×10^6 cells Obtained from 2D flasks	200×10^6 cells Obtained from 2D flasks	40×10^6 cells Obtained from thawed cryovials	200×10^6 cells Obtained from 2D flasks
**Expansion**	1 day attachment + 3 days expansion	1 day attachment + 3 days expansion	1.5 days attachment + 5.5 days expansion	1 day attachment + 3 days expansion
**Transfection**	N/A	2D historical parameters	N/A	2D historical parameters (PEI:DNA ratio changed from 3:1 to 1:1)
**Production**	N/A	2 days production	N/A	2 days production
**Harvest**	Release Adherent Cells and Harvest	Release Adherent Cells and Harvest	Release Adherent Cells and Harvest	Release Adherent Cells and Harvest
**Lysis**	N/A	Historical lysis (at 2×10^6 cells/mL) performed in a 2D flask.	N/A	Historical lysis (at 2×10^6 cells/mL) performed in a 2D flask.

Table S8: Description of the 3 Quantum® runs used to develop the in-situ lysis process step.

Process step	5th run	6th run	7th run
**Seeding**	$200x10^6$ cells<br>Obtained from 2D flasks	$40x10^6$ cells<br>Obtained from thawed cryovials	$40x10^6$ cells<br>Obtained from thawed cryovials
**Expansion**	1 day attachment + 4 days expansion	1.5 days attachment + 5.5 days expansion	1.5 days attachment + 5.5 days expansion
**Transfection**	Optimised parameters	Optimised parameters	Optimised parameters
**Production**	2 days production	2 days production	2 days production
**Harvest**	N/A	N/A	Release Adherent Cells, no Harvest
**Lysis**	Historical lysis, buffer introduced via reagent line	DoE at small scale on harvest material	POC lysis with bypass, time-course sampling

Table S9: Summary of yields obtained during the 3 Quantum® runs used to determine seeding density thus predicted density at transfection for the engineering runs.

Predicted density at transfection (cells/cm^2)		Yield (total vg)						Lysis recovery (%)	Lysis & supernatants recovery (%)
		24h supernatant	48h supernatant	72h supernatant	Lysate	Flush post-lysis	Total		
**8th run**	**56K**	1.49×10^{12}	2.39×10^{12}	8.71×10^{11}	5.62×10^{13}	2.94×10^{12}	6.39×10^{13}	61.6	95.4
**10th run**	**83.3K**	2.31×10^{11}	1.12×10^{11}	4.25×10^{10}	1.56×10^{14}	1.84×10^{12}	1.58×10^{14}	98.7	99.0
**9th run**	**110K**	2.22×10^{12}	5.00×10^{12}	2.07×10^{12}	3.18×10^{13}	6.57×10^{12}	4.76×10^{13}	66.8	86.3
